# Large-scale Genomic Landscape and Clinical Outcomes of De Novo and Treatment-emergent Neuroendocrine Prostate Cancer

**DOI:** 10.1016/j.euros.2026.06.003

**Published:** 2026-07-02

**Authors:** Kazuki Iida, Kojiro Tashiro, Fumihiko Urabe, Yuya Matsui, Yusei Urabe, Takaaki Ishikawa, Juntaro Matsuzaki, Kentaro Yoshihara, Takahiro Kimura, Yoshimasa Saito

**Affiliations:** a Division of Pharmacotherapeutics, Keio University Faculty of Pharmacy, Tokyo, Japan; b Department of Urology, The Jikei University School of Medicine, Tokyo, Japan; c Division of Interdisciplinary Genetics and Nanomedicine, Research Center for Drug Discovery, Keio University Faculty of Pharmacy, Tokyo, Japan; d Division of Gastroenterology and Hepatology, Department of Internal Medicine, Keio University School of Medicine, Tokyo, Japan

**Keywords:** Neuroendocrine prostate cancer (NEPC), de novo NEPC, Treatment-emergent NEPC (t-NEPC), *TP53*, *RB1*, *PTEN*

## Abstract

**Background and objective:**

Neuroendocrine prostate cancer (NEPC) is a rare, aggressive subtype of prostate cancer that arises either as de novo or as a treatment-emergent phenotype during androgen-deprivation therapy. Given its rarity, large-scale studies comparing genomic features and clinical outcomes between de novo NEPC and treatment-emergent NEPC (t-NEPC) are limited. We leveraged a nationwide comprehensive genomic profiling database to characterize the genomic landscape of NEPC and to describe the overall survival (OS) after initiation of NEPC treatment, with exploratory comparisons between de novo NEPC and t-NEPC.

**Design, outcome measurements and statistical analysis:**

We retrospectively analyzed 302 patients with NEPC identified from among 5893 patients with prostate cancer registered in the Center for Cancer Genomics and Advanced Therapeutics database. Patients were classified as de novo NEPC (*n* = 184) or t-NEPC (*n* = 118). Genomic alterations detected by comprehensive profiling, including pathogenic/likely pathogenic mutations and oncogenic/likely oncogenic variants, were analyzed. OS was assessed using the Kaplan-Meier method, and prognostic factors were evaluated using Cox proportional hazards models.

**Results and limitations:**

The most frequent genomic alterations in de novo NEPC and t-NEPC, respectively, were *TP53* (50% and 49%), *RB1* (44% and 44%), *PTEN* (21% and 24%), *BRCA2* (15% and 23%), and *MYC* (17% and 14%), with no statistically significant differences across histology and specimen site. Median OS from initiation of NEPC treatment was 21.5 mo (95% confidence interval [CI], 15.6–32.1) in de novo NEPC and 13.6 mo (95% CI, 11.5–32.2) in t-NEPC, corresponding to a median difference of 7.9 mo (bootstrap 95% CI, 3.1–13.7); however, the between-group difference was not statistically significant (log-rank *p* = 0.3; hazard ratio, 1.29 [95% CI, 0.81–2.07]). In exploratory multivariable analysis, concurrent alterations in *TP53*, *RB1*, and *PTEN* were statistically significantly associated with a higher hazard of death after initiation of NEPC treatment compared with no alterations in these genes.

**Conclusions:**

In this nationwide genomic analysis, de novo NEPC and t-NEPC showed largely similar genomic profiles after neuroendocrine differentiation; however, these findings should be interpreted with caution because of limitations in available clinical variables. These findings provide real-world insights into the clinical and genomic characteristics of NEPC.

## Take Home Message

This nationwide real-world study reveals that de novo and treatment-emergent neuroendocrine prostate cancer share similar genomic landscapes. Concurrent *TP53*, *RB1*, and *PTEN* alterations define a subgroup with shorter survival, highlighting an urgent need for genomically informed therapies.

ADVANCING PRACTICE **What does this study add?**

This nationwide real-world study provides the largest integrated genomic and clinical analysis of neuroendocrine prostate cancer (NEPC), comparing de novo and treatment-emergent disease. Despite distinct clinical contexts, de novo NEPC and treatment-emergent NEPC exhibited largely similar genomic landscapes after neuroendocrine differentiation. Alterations in *TP53, RB1*, and *PTEN*—particularly concurrent alterations involving these genes—were associated with a higher hazard of death, suggesting the presence of a genomically defined higher-risk subgroup. These findings suggest that genomic profiling may help refine prognostic stratification and support further investigation of biology-driven therapeutic approaches in NEPC.

**Clinical Relevance**

Neuroendocrine prostate cancer remains a highly aggressive disease with poor survival outcomes, regardless of whether it arises de novo or as treatment-emergent disease. This nationwide analysis demonstrates that de novo NEPC and treatment-emergent NEPC share largely similar genomic profiles, suggesting common biological mechanisms after neuroendocrine differentiation. Concurrent alterations in *TP53*, *RB1*, and *PTEN* were associated with worse survival and may help identify patients with particularly poor prognosis who could benefit from novel therapeutic strategies and clinical trial enrollment. Associate Editor: Roderick C.N. van den Bergh, MD PhD.

**Patient Summary**

In this study, we analyzed genetic changes and survival outcomes in a large group of patients with a rare and aggressive form of prostate cancer called neuroendocrine prostate cancer. We found that patients shared similar genetic features regardless of how the cancer developed, but those with multiple key gene abnormalities tended to have shorter survival. These results suggest that genetic testing may help identify patients who could benefit from new treatment strategies.

## 1 Introduction

Prostate cancer is the most common solid malignancy among men worldwide [1]. Androgen-deprivation therapy (ADT) remains the cornerstone of treatment for advanced prostate cancer and is initially highly effective; however, most of the patients ultimately develop castration-resistant prostate cancer (CRPC), which is associated with disease progression and limited survival benefits. Among the heterogeneous spectrum of CRPC, neuroendocrine prostate cancer (NEPC) represents a rare but highly aggressive subtype, accounting for ∼1–2% of all prostate cancers. NEPC is characterized by rapid disease progression, visceral metastases, low prostate-specific antigen (PSA) levels, and an extremely poor prognosis, with a reported median survival of <1 yr [2], [3].

Clinically, NEPC is broadly classified into two entities: de novo NEPC, which is present at the time of initial diagnosis, and treatment-emergent NEPC (t-NEPC), which develops during the course of ADT as a therapy-resistant phenotype. t-NEPC is thought to arise through lineage plasticity under selective pressure from androgen receptor (AR)–targeted therapies and has been reported to occur in ∼15–30% of metastatic CRPC cases [4]. In contrast, de novo NEPC is exceedingly rare, accounting for <1% of all prostate cancers. Despite these distinct clinical contexts, the biological mechanisms driving NEPC development as well as the molecular similarities and differences between de novo NEPC and t-NEPC remain poorly understood. Moreover, effective therapeutic options for both entities are limited [5], [6].

Given its pathological and molecular resemblance to small-cell lung cancer (SCLC), platinum-based chemotherapy regimens commonly used for SCLC have been empirically adopted for patients with NEPC [7], [8]. Nevertheless, treatment responses are often transient, and outcomes remain dismal. Owing to the rarity of NEPC, most prior studies have been limited to small institutional cohorts or pathological case series, and comprehensive large-scale genomic analyses incorporating clinical outcomes are scarce. As a result, the prognostic relevance of specific genomic alterations and their potential implications for therapeutic stratification in NEPC have not been fully elucidated.

In Japan, comprehensive genomic profiling (CGP) has been reimbursed under the national health insurance system since 2019, enabling systematic molecular characterization of advanced solid tumors in routine clinical practice. All resulting clinical and genomic data are centrally aggregated in the Center for Cancer Genomics and Advanced Therapeutics (C-CAT) database [9]. This nationwide real-world database provides a unique opportunity to investigate rare malignancies such as NEPC at an unprecedented scale. Therefore, the aims of this study were as follows: (1) to describe the genomic alteration profile of NEPC in a nationwide C-CAT cohort, (2) to compare genomic alteration frequencies descriptively between de novo NEPC and t-NEPC and across selected clinicopathological subgroups, and (3) to evaluate overall survival (OS) after initiation of NEPC treatment and its exploratory associations with key genomic alterations.

## 2 Materials and methods

### 2.1 Study design and patients

**Fig. 1:**
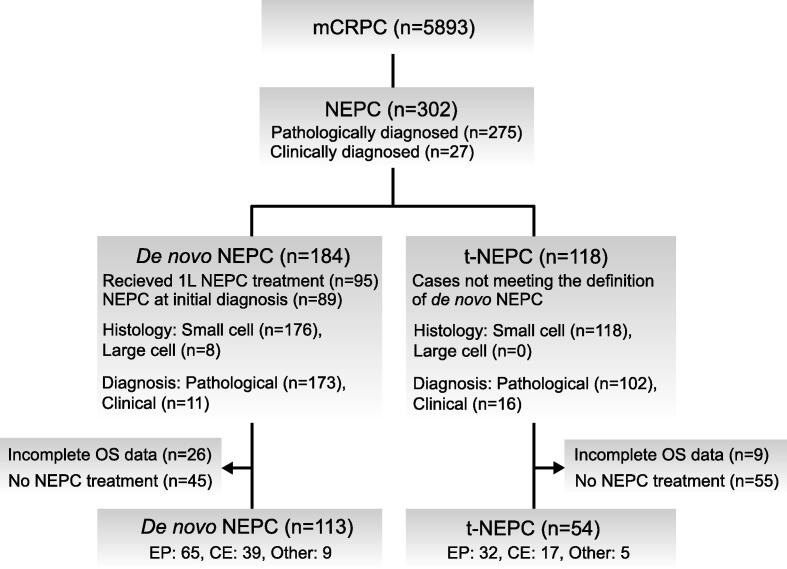
Flow diagram and cohort characteristics of patients with NEPC. Flow diagram illustrating patient selection, classification, diagnostic confirmation status, histology, and inclusion in the survival analysis. A total of 5893 patients with mCRPC were reviewed, of whom 302 met the study definition of NEPC and were classified as de novo NEPC (*n* = 184) or t-NEPC (*n* = 118). Among them, 167 patients with documented NEPC treatment initiation and follow-up data were included in the survival analysis, comprising 113 with de novo NEPC and 54 with t-NEPC. 1L = first-line; CE = etoposide + carboplatin; EP = etoposide + cisplatin; mCRPC = metastatic castration-resistant prostate cancer; NEPC = neuroendocrine prostate cancer; OS = overall survival; t-NEPC = treatment-emergent neuroendocrine prostate cancer.

From the C-CAT database, a total of 5893 patients with prostate cancer for whom CGP data were available were reviewed, of which 302 patients diagnosed with NEPC were included in the analysis, including 184 with de novo NEPC and 118 with t-NEPC. These data were derived from records registered in the C-CAT database between June 2019 and June 2025. A detailed flow diagram illustrating patient selection and categorization is presented in Fig. 1. Baseline clinical characteristics were obtained from the information registered in the C-CAT database. Performance status was assessed using the Eastern Cooperative Oncology Group scale as recorded in the database. This study was reviewed and approved by the institutional review board of Keio University (Tokyo, Japan) and the C-CAT Data Utilization Review Board (Approval ID: CDU2023-039).

### 2.2 NEPC type

Using the C-CAT database, patients with NEPC were identified based on their diagnostic information. Eligible patients included those with a registered diagnosis of neuroendocrine carcinoma and those with a pathological or clinical diagnosis containing the terms “neuroendocrine prostate cancer,” “prostate small-cell carcinoma,” “small-cell neuroendocrine prostate carcinoma,” or “large-cell neuroendocrine prostate carcinoma.” Based on these criteria, 302 patients were included in the analysis.

Using pathological or clinical diagnoses, CGP registration date, specimen collection date for CGP, and types of therapeutic agents administered, patients were classified as de novo NEPC (*n* = 184) or t-NEPC (*n* = 118). From a pathological perspective, tumors were categorized into two major histological subtypes: small-cell NEPC (SCNEPC) and large-cell NEPC (LCNEPC). Patients described as having mixed neuroendocrine carcinoma were classified as SCNEPC unless otherwise specified.

De novo NEPC was defined as patients who met the following criteria:

1. NEPC treatment was administered as the first-line systemic treatment (de novo NEPC1).
2. NEPC treatment was not administered as the first-line systemic treatment, but biopsy specimens obtained at initial diagnosis were judged to contain NEPC components, or those with an interval of ≤30 d between the date of prostate cancer diagnosis and the specimen collection date submitted for CGP as NEPC, suggesting that NEPC was present at the time of initial prostate cancer diagnosis (de novo NEPC2).

Among patients classified as having de novo NEPC, 11 were diagnosed clinically and had initiated NEPC treatment without pathological confirmation at diagnosis.

All remaining patients with a pathological or clinical diagnosis of NEPC were classified as t-NEPC. Among patients classified as having t-NEPC, 102 were confirmed pathologically, and 16 patients diagnosed on clinical grounds were also included in the analysis. The definition of clinical diagnosis in t-NEPC was based on clinical course and treatment history rather than mandatory histological confirmation of neuroendocrine transformation. In this cohort, most of the patients with NEPC (275/302, 91%) were pathologically confirmed, whereas a minority were classified as having clinically diagnosed NEPC. Because detailed tumor marker data, including PSA and neuroendocrine markers, were not available in the C-CAT database, clinical diagnosis could not be established based on predefined biomarker criteria. Instead, patients classified as having clinically diagnosed NEPC were identified based on the diagnostic labels recorded in the registry, together with a structured assessment of available clinical information, including the date of prostate cancer diagnosis, timing of initiation of NEPC treatment, CGP registration date, specimen collection date, and prior treatment history. These variables were used to assess whether the clinical course was consistent with NEPC as recorded in the registry. No additional reclassification beyond the information recorded in the registry was performed.

### 2.3 NEPC treatment

*NEPC treatment* was defined a priori as systemic chemotherapy administered with the intent to treat NEPC based on treatment paradigms commonly used for small-cell carcinoma/high-grade neuroendocrine carcinoma. NEPC treatment included platinum-etoposide combinations, cisplatin + etoposide and carboplatin + etoposide, as well as other regimens used in neuroendocrine/small-cell settings, including carboplatin + irinotecan, cisplatin + irinotecan, etoposide monotherapy, irinotecan monotherapy, nogitecan, amrubicin, and carboplatin + paclitaxel.

This classification was selected because platinum-etoposide is widely regarded as a core regimen for small-cell carcinoma/high-grade neuroendocrine carcinoma. In addition, several included agents/regimens, such as platinum-irinotecan, and topoisomerase inhibitors, such as topotecan/nogitecan, as well as amrubicin, are established or commonly used treatments for SCLC and have also been applied in published treatment series of t-NEPC, supporting their inclusion as NEPC treatment in this study.

### 2.4 Gene alteration

Tumor gene profiling was performed by next-generation sequencing using one of the five approved CGP tests. Results were reviewed by a molecular tumor board (MTB) comprising oncologists, geneticists, and other specialists [10]. The classification of variant pathogenicity followed the definitions of the American College of Medical Genetics and Genomics. Only variants categorized as “pathogenic,” “likely pathogenic,” “oncogenic,” or “likely oncogenic” were included, whereas variants of uncertain significance or benign were excluded. Genetic alterations were classified into four categories—mutation, loss, amplification, and rearrangement—and separate OncoPrints were generated for de novo NEPC and t-NEPC. Furthermore, based on our analysis, the top 10 recurrently altered genes (*TP53*, *RB1*, *PTEN*, *BRCA2*, *MYC*, *TMPRSS2*, *ERG*, *KMT2D*, *CDK12*, and *SPEN*) were selected. Alteration frequencies of these genes were compared according to NEPC type (de novo NEPC vs t-NEPC), specimen site (primary vs metastatic lesion), histology (SCNEPC vs LCNEPC), and specimen type (liquid biopsy, surgical specimen, or tumor biopsy).

### 2.5 Statistical analysis

The primary objective of this study was to descriptively characterize the genomic landscape of NEPC, including comparisons of alteration frequencies between de novo NEPC and t-NEPC, as well as across selected clinicopathological subgroups. Secondary exploratory objectives were to describe OS after initiation of NEPC treatment and to evaluate associations between key genomic alterations and OS.

For descriptive analyses of the study cohort, continuous variables are summarized as median and interquartile range (IQR) and categorical variables as number and percentage. Baseline characteristics were compared descriptively between de novo NEPC and t-NEPC using the Wilcoxon rank-sum test for continuous variables and the Fisher exact test for categorical variables.

For genomic analyses, alteration frequencies of selected recurrent genes were summarized according to NEPC type, specimen site, histology, and specimen type. For these descriptive comparisons, genomic alterations were categorized by alteration class (eg, mutation and copy number alteration [CNA]) where applicable, and subgroup comparisons were performed using the Fisher exact test. Because these subgroup comparisons were intended to describe frequency patterns rather than formally test effect modification, they were considered exploratory.

In the C-CAT database, the exact date of NEPC diagnosis, particularly for patients classified as t-NEPC, was often unavailable. Therefore, the date of initiation of NEPC treatment (eg, etoposide + cisplatin or carboplatin) was used as the index date for the primary survival analyses. This time point was used as a surrogate because alternative time points, including the precise date of pathological diagnosis, could not be reliably defined even among pathologically confirmed patients with t-NEPC, and the overall cohort included both pathologically confirmed and clinically diagnosed patients.

OS from the initiation of NEPC treatment (*OS from NEPC treatment*) was also defined as the time from initiation of first-line NEPC treatment to death from any cause. Survival status and dates of last confirmed survival were obtained from longitudinal clinical information registered in the C-CAT database based on visit dates recorded in the registry. Patients without a recorded death were censored at the date of last confirmed survival. Median follow-up was estimated using the reverse Kaplan-Meier method, with follow-up defined from initiation of NEPC treatment to death or last confirmed survival. Patients who did not receive NEPC treatment, had insufficient data on the date of treatment initiation, or lacked a recorded date of last confirmed survival were excluded from the survival analysis. Kaplan-Meier methods were used to estimate OS, and survival curves were compared using the log-rank test. Kaplan-Meier curves were truncated at the first prespecified 5-mo interval at which the number at risk in any displayed group fell below five. The 95% confidence interval (CI) for the difference in median OS between de novo NEPC and t-NEPC was estimated using stratified patient-level bootstrap resampling by NEPC type with 5000 iterations and the percentile method.

To visualize treatment trajectories before and after initiation of NEPC treatment, Sankey diagrams were generated separately for de novo NEPC and t-NEPC among patients who received NEPC treatment. These plots were descriptive and used the line of therapy as the horizontal framework to summarize treatment flow.

To explore prognostic associations, Cox proportional hazards regression models were used to evaluate the associations of *TP53*, *RB1*, and *PTEN* alterations as well as concurrent alterations in these genes, with OS from NEPC treatment. Multivariable models were adjusted for age at registration, performance status, and metastatic pattern at registration. Exploratory interaction models between NEPC type and genomic alteration status were also fitted for *TP53*, *RB1*, and *PTEN* alterations as well as for the *TP53*/*RB1*/*PTEN* alteration group. The primary analyses used restricted follow-up with administrative censoring at the prespecified truncation time point, and full follow-up analyses were performed as secondary analyses.

All statistical analyses were performed using R software version 4.5.0 (R Foundation for Statistical Computing, Vienna, Austria). All tests were two sided, and *p* values <0.05 were considered statistically significant.

## 3 Results

### 3.1 Study population

Among a total of 5893 patients with prostate cancer in the C-CAT database, 302 patients met the criteria for NEPC as defined in this study, including 184 with de novo NEPC and 118 with t-NEPC (Fig. 1). Within the de novo NEPC group, 176 patients were classified as having SCNEPC and eight as having LCNEPC. Among patients with t-NEPC, all 118 were classified as having SCNEPC, with no patients with LCNEPC identified (Fig. 1). Baseline characteristics of patients with de novo NEPC and t-NEPC are summarized in Supplementary Table 1. In patients with t-NEPC, MTB discussions occurred more frequently at later lines of treatment, and liquid biopsy specimens were used more often. Pathological confirmation of NEPC was unavailable in 11 patients with de novo NEPC and 16 patients with t-NEPC; in these patients, NEPC was diagnosed based on clinical judgment integrating disease course, treatment history, and available clinical information (Fig. 1).

### 3.2 Genomic landscape of NEPC

**Fig. 2:**
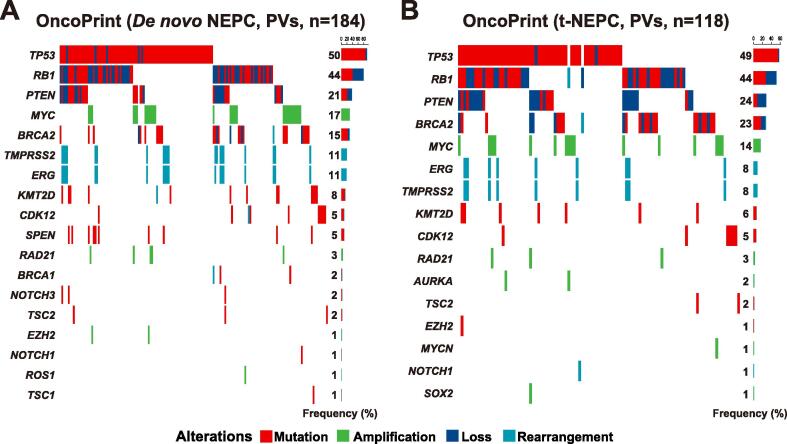
OncoPrint summarizing the genomic landscape of de novo NEPC and t-NEPC. (A) OncoPrint of de novo NEPC (*n* = 184). (B) OncoPrint of t-NEPC (*n* = 118). The bar plots show the frequencies of recurrent alterations, and alterations are categorized as mutation, amplification, loss, and rearrangement. NEPC = neuroendocrine prostate cancer; PV = pathogenic variant; t-NEPC = treatment-emergent neuroendocrine prostate cancer.

The overall genomic alterations identified in NEPC are summarized in the OncoPrint (Fig. 2). Across both de novo NEPC and t-NEPC, respectively, the most frequent alterations were *TP53* (50% and 49%), *RB1* (44% and 44%), *PTEN* (21% and 24%), *BRCA2* (15% and 23%), and *MYC* (17% and 14%), followed by *ERG*, *TMPRSS2*, *KMT2D*, and *CDK12*. To address potential diagnostic misclassification, we analyzed genomic alterations in both pathologically confirmed and clinically diagnosed patients with NEPC. The overall alteration patterns, including the frequencies of *TP53*, *RB1*, and *PTEN* alterations, were comparable regardless of whether patients were pathologically confirmed or clinically diagnosed across de novo NEPC and t-NEPC (Supplementary Fig. 1).

We further compared alteration frequencies of highly prevalent genes by NEPC type (de novo NEPC vs t-NEPC), specimen site (primary vs metastatic lesions), histology (SCNEPC vs LCNEPC), and specimen type (tumor biopsy, surgical specimen, or liquid biopsy). Overall, patterns of genomic alterations were largely similar across subgroups, with no statistically significant differences observed for most genes. However, the detection rates of *TP53* mutations, *RB1* loss, and *RB1* alterations varied by specimen type (Supplementary Fig. 2).

To further characterize the cohort, treatment trajectories for the 202 patients who received NEPC treatment are shown in a Sankey diagram stratified by NEPC type (de novo NEPC vs t-NEPC; Supplementary Fig. 3). We also show the treatment flow according to the predefined subcategories of de novo NEPC (de novo NEPC1 and de novo NEPC2; Supplementary Fig. 4).

### 3.3 Oncological outcomes in patients with NEPC

Among the 302 patients with NEPC, 167 patients (113 de novo NEPC and 54 t-NEPC) with documented dates of NEPC treatment initiation were included in the OS analysis. The median follow-up from initiation of NEPC treatment was 20 mo (95% CI, 16–25). Baseline characteristics of patients with de novo NEPC and t-NEPC in this cohort are summarized in Supplementary Table 2. Among patients with t-NEPC, 57% (31/54) had received two or more systemic therapies before NEPC treatment, indicating frequent exposure to multiple treatment lines before neuroendocrine transformation. The median time from initial prostate cancer diagnosis to t-NEPC treatment was 21 mo (IQR, 10–40). In the de novo NEPC group, 36% (41/113) of the patients received non-NEPC treatment as first-line treatment, despite a diagnosis of NEPC at presentation.

Among the 167 patients evaluable for post-NEPC treatment (or treatment at diagnosis for de novo NEPC), first-line therapies were predominantly etoposide + cisplatin (*n* = 97, 58%) or etoposide + carboplatin (*n* = 56, 34%). In addition, 12 patients received other platinum-based regimens (eg, irinotecan + cisplatin), whereas the remaining two patients received nonplatinum regimens consisting of etoposide monotherapy and amrubicin monotherapy, respectively. Overall, 165 of the 167 patients (99%) received a platinum-based regimen as first-line NEPC treatment (Fig. 1).

**Fig. 3:**
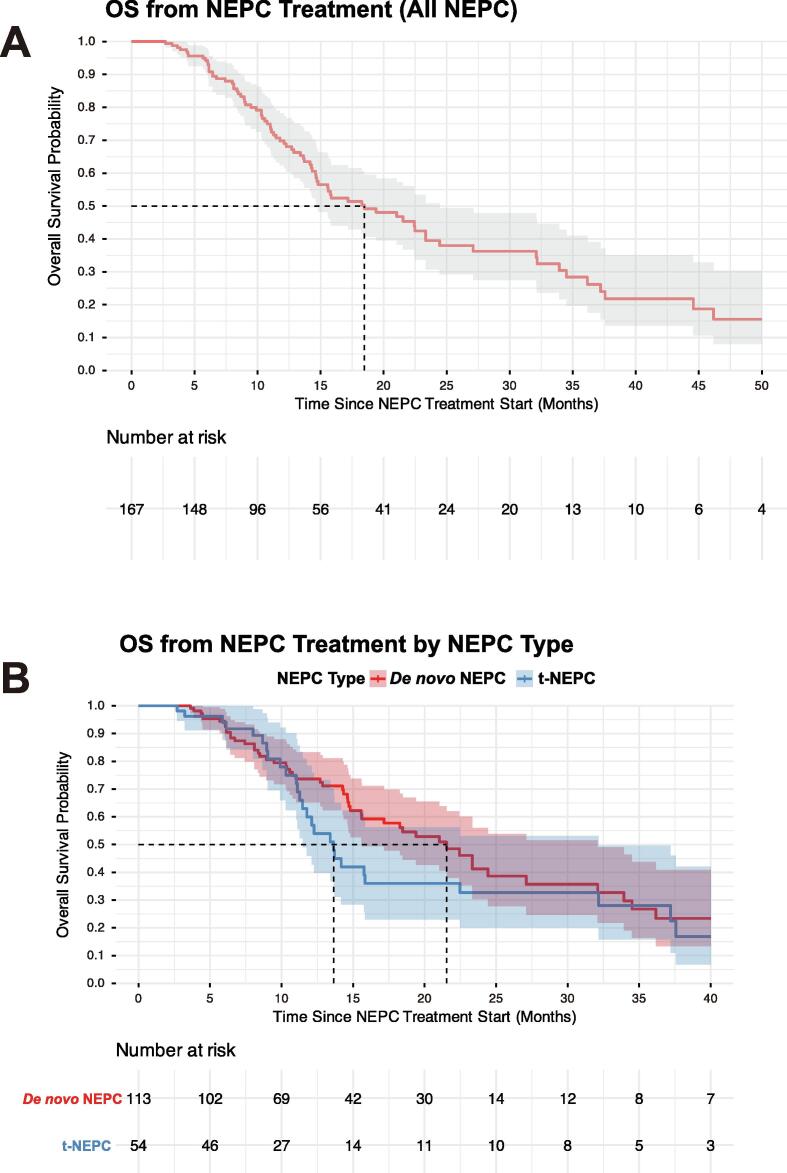
Survival analysis of patients with NEPC. (A) Kaplan-Meier curves for OS from initiation of NEPC treatment in all patients included in the prognostic analysis. (B) Kaplan-Meier curves for OS from initiation of NEPC treatment according to NEPC type. *Time zero* was defined as the date of initiation of first-line NEPC treatment. NEPC = neuroendocrine prostate cancer; OS = overall survival. See also Supplementary Table 3 for statistical details.

In the analysis of all patients with NEPC, median *OS from NEPC treatment* was 18.5 mo (95% CI, 14.7–24.4; Fig. 3A; Supplementary Table 3). Median *OS from NEPC treatment* was 21.5 mo (95% CI, 15.6–32.1) in de novo NEPC and 13.6 mo (95% CI, 11.5–32.2) in t-NEPC, corresponding to a median difference of 7.9 mo (bootstrap 95% CI, 3.1–13.7); however, the between-group difference was not statistically significant (log-rank *p* = 0.3; hazard ratio [HR], 1.29 [95% CI, 0.81–2.07]) as shown in Fig. 3B and Supplementary Table 3. We performed a similar analysis of OS restricted to pathologically confirmed patients with NEPC and confirmed that the results were consistent with those of the overall cohort (Supplementary Fig. 5; Supplementary Table 8).

We also analyzed *OS from NEPC treatment* by de novo NEPC subtype (de novo NEPC1 and de novo NEPC2) and compared the results with those in t-NEPC. There was no statistically significant difference in *OS from NEPC treatment* between de novo NEPC1 and de novo NEPC2, and comparisons with t-NEPC showed similar results to those presented in Fig. 3 (Supplementary Fig. 6; Supplementary Table 9).

### 3.4 Oncological outcomes according to genomic alterations in NEPC

**Fig. 4:**
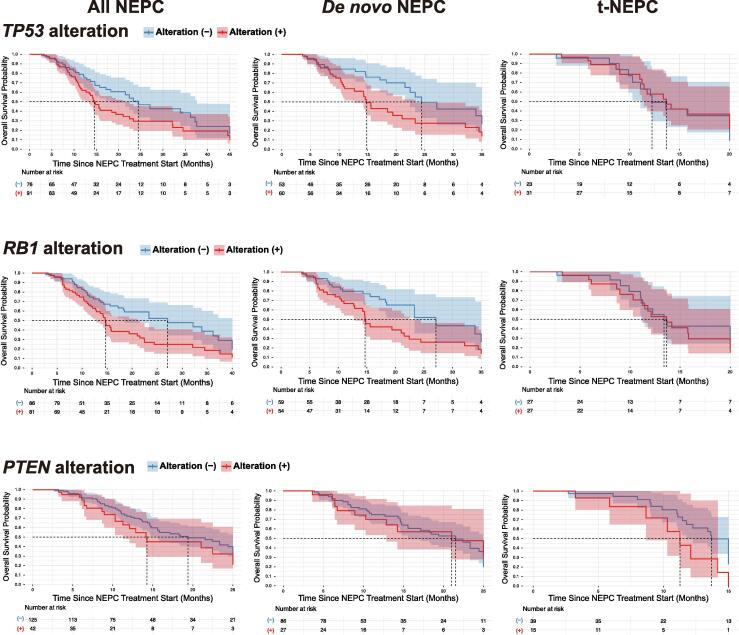
Impact of *TP53*, *RB1*, and *PTEN* alterations on OS from NEPC treatment in patients with NEPC. Kaplan-Meier curves for OS from initiation of NEPC treatment stratified by the presence or absence of *TP53*, *RB1*, and *PTEN* alterations. Analyses are shown for all patients with NEPC (left panels), de novo NEPC (middle panels), and t-NEPC (right panels). NEPC = neuroendocrine prostate cancer; OS = overall survival; t-NEPC = treatment-emergent neuroendocrine prostate cancer. See also Supplementary Table 4 for statistical details.

*OS from NEPC treatment* was compared between the de novo NEPC and t-NEPC groups according to the presence or absence of key genomic alterations, including *TP53*, *RB1*, and *PTEN*. In the overall NEPC cohort, *TP53* and *RB1* alterations were statistically significantly associated with a higher hazard of death after initiation of NEPC treatment (Fig. 4; Supplementary Table 4). *PTEN* alteration was not significantly associated with OS in the overall cohort, although the point estimate suggested a higher hazard of death. These associations were broadly similar in de novo NEPC. In t-NEPC, *TP53* and *RB1* alterations were not significantly associated with OS, whereas *PTEN* alteration was statistically significantly associated with a higher hazard of death; these subgroup findings should be interpreted as exploratory (Fig. 4; Supplementary Table 4). We performed a similar analysis in pathologically confirmed patients with NEPC and confirmed that the results were consistent with those of the overall cohort (Supplementary Fig. 7; Supplementary Table 10).

**Fig. 5:**
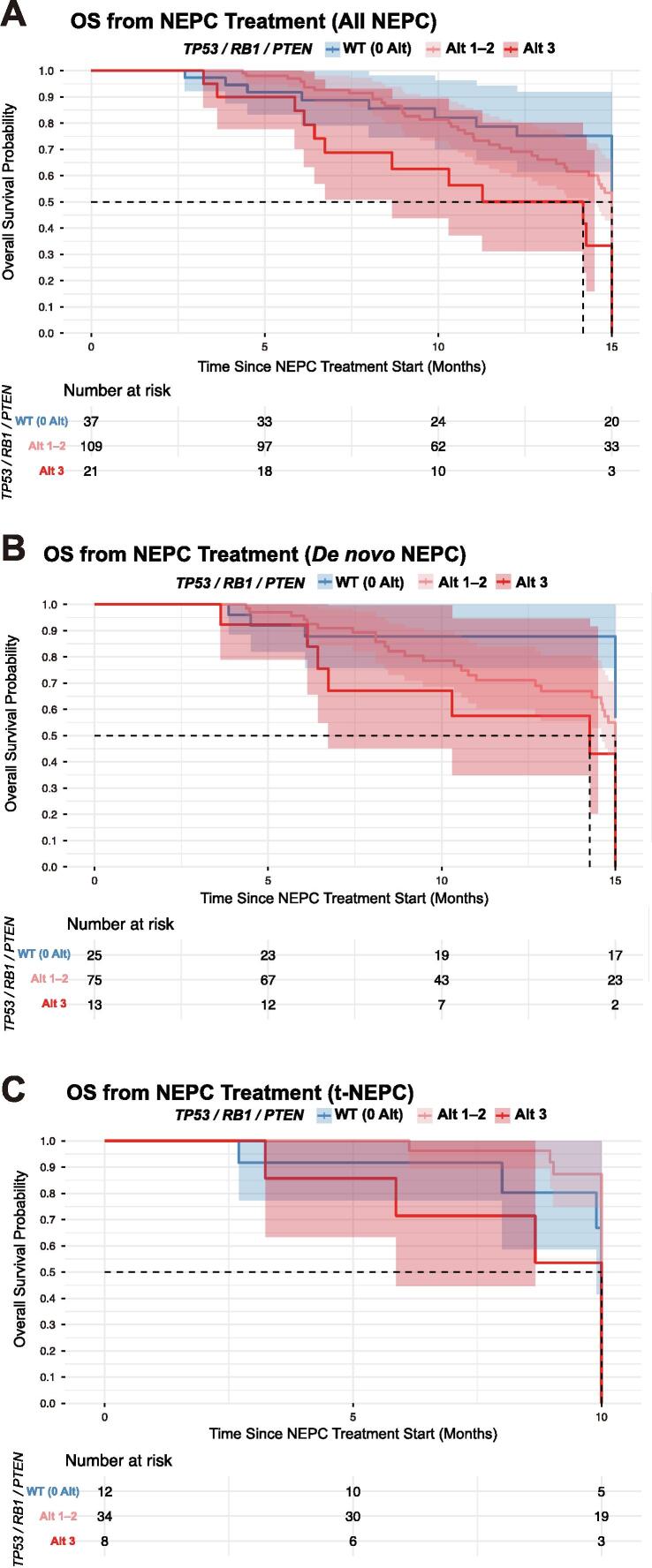
Impact of combined *TP53*, *RB1*, and *PTEN* alterations on OS from NEPC treatment in patients with NEPC. Kaplan-Meier curves showing OS from initiation of NEPC treatment, stratified by the number of altered genes among *TP53*, *RB1*, and *PTEN* (zero, one to two, or three alterations). Curves were truncated at the prespecified time point when the number at risk became low. (A) All patients with NEPC. (B) Patients with de novo NEPC. (C) Patients with t-NEPC. NEPC = neuroendocrine prostate cancer; OS = overall survival; t-NEPC = treatment-emergent neuroendocrine prostate cancer. See also Supplementary Table 5–7 for statistical details.

We further evaluated the association of concurrent alterations in *TP53*, *RB1*, and *PTEN* and *OS from NEPC treatment*. In the overall NEPC cohort, the triple-alteration group was statistically significantly associated with a higher hazard of death after initiation of NEPC treatment compared with the *wild-type group*, defined as no alterations in these three genes, in the restricted follow-up analysis (HR, 3.47 [95% CI, 1.67–7.23]; Cox *p* < 0.001; Fig. 5A; Supplementary Table 5). When stratified by disease subtype, evidence of differences across the three alteration groups was observed in de novo NEPC and t-NEPC in the restricted follow-up analysis (global log-rank *p* = 0.01 and *p* = 0.03, respectively). In both subtypes, the HR for the triple-alteration group compared with the wild-type group was >1, although the estimates were imprecise, particularly in t-NEPC (de novo NEPC: HR, 4.40 [95% CI, 1.65–11.71]; t-NEPC: HR, 1.72 [95% CI, 0.58–5.14]; Fig. 5B and 5C; Supplementary Table 5). In the secondary full follow-up analysis, the corresponding global log-rank test in t-NEPC was not statistically significant (Supplementary Table 5).

In multivariable Cox regression analysis using restricted follow-up, concurrent *TP53*, *RB1*, and *PTEN* alterations remained statistically significantly associated with a higher hazard of death after initiation of NEPC treatment in the overall NEPC cohort compared with no alterations in these genes (adjusted HR, 3.08 [95% CI, 1.20–7.88]; Cox *p* = 0.02; Supplementary Table 6).

Subgroup-specific estimates were imprecise, particularly in t-NEPC. These analyses were exploratory and based on the variables available in the C-CAT database, and residual confounding could not be excluded. Broadly similar trends were observed in patients with pathologically confirmed NEPC (Supplementary Fig. 8; Supplementary Table 11).

In exploratory interaction analyses, only the NEPC type and *PTEN* alteration interaction were statistically significant in the primary restricted follow-up analysis (adjusted interaction HR, 3.32 [95% CI, 1.04–10.61]; *p* = 0.043), whereas this interaction was not statistically significant in the secondary full follow-up analysis (Supplementary Table 7). These findings should be interpreted cautiously because of the limited subgroup sample sizes.

## 4 Discussion

In this nationwide study using the C-CAT database, we characterized the genomic landscape of 302 Japanese patients with NEPC, stratified into de novo NEPC and t-NEPC, and evaluated treatment outcomes within each subgroup. To the best of our knowledge, this study represents one of the largest real-world clinical genomic analyses of NEPC to date. Notably, no prior investigation has systematically examined the molecular features of NEPC and their clinical relevance at this scale, positioning this study as the first nationwide, real-world genomic-clinical assessment of NEPC. The proportion of de novo NEPC in our cohort appears higher than that reported in epidemiological studies, where de novo NEPC is considered rare. This discrepancy likely reflects selection bias inherent to the C-CAT registry, which includes patients undergoing CGP, typically those with advanced or treatment-refractory disease. Furthermore, this registry includes both pathological and clinical diagnoses of NEPC, which may capture a subset of tumors with aggressive clinical or molecular features or neuroendocrine differentiation that prompted clinicians to initiate NEPC treatment. Accordingly, some clinically diagnosed patients may have had aggressive variant adenocarcinoma rather than true neuroendocrine carcinoma. In addition, the definition of t-NEPC based on clinical course rather than histological confirmation may have introduced biological heterogeneity when comparing de novo NEPC and t-NEPC.

Given the rarity of NEPC, large-scale studies remain scarce. Most previous reports have been limited by small sample sizes, and even relatively larger studies have primarily focused on genomic profiling without incorporating survival analyses [11], [12]. A recent meta-analysis reported that *TP53* mutations (49%) and CNAs, including *RB1* loss (58%), *TP53* loss (43%), *PTEN* loss (37%), *AURKA* amplification (28%), and *MYCN* amplification (23%), were among the most frequently observed genomic events in NEPC [13]. Consistently, *TP53* alterations were identified as the most prevalent genomic event in our cohort, occurring at similar frequencies in de novo NEPC and t-NEPC (50% vs 49%), followed by *RB1* alterations (44% vs 44%), *PTEN* alterations (21% vs 24%), *BRCA2* alterations (15% vs 23%), and *MYC* amplifications (17% vs 14%). Overall, the genomic profiles of de novo NEPC and t-NEPC were largely similar, and the genomic alteration spectrum observed in our dataset showed no major discrepancies compared with the previous meta-analysis [13].

In our genomic profiling–based survival analysis, we focused on alterations in *TP53*, *RB1*, and *PTEN*, which were among the most frequently altered genes in NEPC. Both *TP53* and *RB1* are tumor suppressor genes involved in cell-cycle regulation, deoxyribonucleic acid (DNA) damage response, and maintenance of cellular differentiation [14], [15]. The loss of *TP53* disrupts DNA damage response and apoptotic pathways, enabling the survival of aberrant cells. Loss of *RB1* leads to activation of E2F transcription factors, which in turn promote cell-cycle progression and expression of stemness-associated genes such as *SOX2* and *EZH2* [16]. The concurrent loss of both genes induces genomic instability and triggers lineage plasticity, thereby facilitating the transdifferentiation of prostate adenocarcinoma cells from an AR–dependent luminal phenotype to an AR-independent neuroendocrine phenotype [17]. Additionally, *PTEN*, a key tumor suppressor gene that negatively regulates the phosphatidylinositol-3-kinase/AKT signaling pathway, is among the most frequently altered genes in advanced prostate cancer [18]. Loss of *PTEN*, particularly when co-occurring with *TP53* or *RB1* alterations, has been implicated in disease progression, metastatic potential, and resistance to ADT [19]. Enhanced neuroendocrine differentiation has also been observed in mouse models with combined knockdown of *RB1*, *TP53*, and *PTEN* [19]. Moreover, alterations in *TP53*, *RB1*, and *PTEN* have been reported in cohorts of aggressive variant prostate cancer, including de novo NEPC and t-NEPC, as well as in metastatic CRPC, where tumors harboring concurrent alterations in two or three of these genes are associated with poorer clinical outcomes [4], [20]. Consistent with these findings, our cohort analysis showed hazard ratio estimates >1 in patients harboring two or more alterations in *TP53*, *RB1*, and *PTEN*, although some subgroup-specific estimates were imprecise.

However, these results should be interpreted with caution. Although these genomic alterations are well-recognized phenotypic features of high-grade prostate cancer, their clinical utility in the management of NEPC represents an important area for future investigation.

Currently, there is no established standard treatment for NEPC. Owing to its clinical, pathological, and molecular similarities to SCLC, systemic chemotherapy regimens developed for SCLC have been widely adopted in the management of NEPC [21]. According to the National Comprehensive Cancer Network Guidelines for Prostate Cancer, recommended treatment options for NEPC include cisplatin + etoposide, carboplatin + etoposide, docetaxel + carboplatin, and cabazitaxel + carboplatin [22], [23], [24], [25]. In our cohort, > 90% of the patients—regardless of whether they had de novo NEPC or t-NEPC—received etoposide-based platinum chemotherapy (etoposide + cisplatin or carboplatin) as NEPC treatment following neuroendocrine differentiation. In contrast, the median OS following NEPC treatment was 21.5 mo (95% CI, 15.6–32.1) in patients with de novo NEPC and 13.6 mo (95% CI, 11.5–32.2) in those with t-NEPC (log-rank *p* = 0.3; HR, 1.29 [95% CI, 0.81–2.07]). Outcomes in both groups remain suboptimal, underscoring the aggressive clinical behavior of NEPC and the urgent need for developing novel therapeutic strategies beyond conventional cytotoxic chemotherapy.

Importantly, in survival analyses, although no statistically significant difference in OS after NEPC treatment initiation was observed between de novo NEPC and t-NEPC, patients with t-NEPC tended to have shorter survival following NEPC treatment. Notably, *TP53* or *RB1* alterations did not stratify OS among patients with t-NEPC, whereas they retained prognostic relevance in de novo NEPC. Collectively, these findings may suggest potential biological differences between de novo NEPC and t-NEPC. Although inactivation of *TP53* and *RB1* is considered a foundational event underlying lineage plasticity and neuroendocrine differentiation, accumulating evidence suggests that t-NEPC progression is not driven solely by genetic alterations detectable by CGP. Instead, epigenetic mechanisms, including chromatin remodeling, enhancer reprogramming, and DNA methylation, appear to play a critical role in driving phenotypic evolution and therapeutic resistance in t-NEPC [26], [27], [28], [29]. These epigenetic alterations may not be adequately captured by CGP platforms, potentially contributing to the observed differences in oncological outcomes between de novo NEPC and t-NEPC. Further integrative studies incorporating epigenomic and transcriptomic profiling are essential to clarify the distinct molecular drivers of de novo NEPC and t-NEPC and guide the development of more effective, biology-driven therapeutic strategies.

Several points warrant careful interpretation of our findings. In this registry-based analysis, t-NEPC was defined based on clinical course and treatment history rather than mandatory histological confirmation after AR-targeted therapy. Therefore, histological confirmation was not performed for some patients with t-NEPC. This approach reflects real-world clinical practice, where repeat biopsies are not always feasible in advanced disease. In addition, the comparison between de novo NEPC and t-NEPC in this study reflects tumors that have already undergone neuroendocrine differentiation. Accordingly, the genomic similarities observed between the two groups may represent convergent molecular features associated with the neuroendocrine phenotype rather than the mechanisms driving lineage plasticity. Because the exact date of NEPC diagnosis was unavailable for many patients, the initiation of NEPC treatment was used as a surrogate for the diagnosis date. This approach may introduce bias related to physician decision-making and disease severity. In addition, survival estimates may have been influenced by treatment selection and the timing of treatment initiation. However, it should be noted that the sensitivity analysis limited to pathologically confirmed patients yielded results consistent with the primary analysis.

This study has several limitations. First, as a retrospective analysis of a CGP database, it is subject to potential selection bias. Although de novo NEPC is typically reported to account for <1% of prostate cancers, it represented ∼3% of the nationwide CGP-tested prostate cancer population in the C-CAT database (184/5893), suggesting enrichment because of referral/selection for CGP in a setting of limited standard treatment options. Second, key clinical parameters such as Gleason score, TNM classification, tumor burden, and metastatic sites were not consistently available in the database. Therefore, patients with missing or incomplete clinical information were excluded during data processing, which may have influenced the analyses; the observed associations between genomic alterations (including *TP53*, *RB1*, and *PTEN*) and survival should be interpreted with caution and should not be considered as evidence of independent prognostic effects. Third, the CGP platforms were limited to panels covering several hundred representative cancer-related genes, precluding assessment of all genomic alterations. Additionally, some analyses were performed using liquid biopsy samples, which have lower sensitivity for detecting genomic alterations than tumor tissue–based next-generation sequencing. Fourth, survival analyses were restricted to patients who received NEPC treatment, which may have introduced additional selection bias. Given these limitations, the generalizability of our findings may be restricted. Nevertheless, the genomic landscape of NEPC observed in this study was largely consistent with previously reported data. In addition, the effective sample size for survival analysis was limited, and potential bias cannot be excluded due to the exclusion of patients with incomplete clinical data. The primary contribution of this study lies in the scale and nationwide real-world nature of the dataset, rather than the identification of novel biological mechanisms or independent prognostic markers.

## 5 Conclusions

In this study, we characterized the genomic landscape and treatment outcomes of NEPC in a Japanese cohort, stratified into de novo NEPC and t-NEPC. The major genomic alterations identified were largely consistent with those reported in previous studies. No statistically significant difference in OS after NEPC treatment was observed between de novo NEPC and t-NEPC. In contrast, concurrent alterations in key tumor suppressor genes were associated with shorter OS in exploratory analyses. Collectively, these findings underscore the clinical importance of refining therapeutic targets in NEPC and highlight the need for more effective, biologically tailored treatment strategies.

  ***Author contributions***: Yoshimasa Saito had full access to all the data in the study and takes responsibility for the integrity of the data and the accuracy of the data analysis.

  *Study concept and design*: Iida, Tashiro, Urabe, Saito.

*Acquisition of data*: Iida, Saito.

*Analysis and interpretation of data*: Iida, Tashiro, Urabe, Matsui, Saito.

*Drafting of the manuscript*: Iida, Tashiro, Urabe, Matsui.

*Critical revision of the manuscript for important intellectual content*: Urabe, Ishikawa, Matsuzaki, Yoshihara.

*Statistical analysis*: Iida, Matsuzaki.

*Obtaining funding*: Saito.

*Administrative, technical, or material support*: None.

*Supervision*: Kimura, Saito.

*Other* (specify): None.

  ***Financial disclosures:*** Yoshimasa Saito certifies that all conflicts of interest, including specific financial interests and relationships and affiliations relevant to the subject matter or materials discussed in the manuscript (eg, employment/affiliation, grants or funding, consultancies, honoraria, stock ownership or options, expert testimony, royalties, or patents filed, received, or pending), are the following: None.

  ***Funding/Support and role of the sponsor*:** This work was supported by the Japan Society for the Promotion of Science (grant numbers: JP24KK0207 and JP24K03285) awarded to Yoshimasa Saito.

  ***Acknowledgments:*** The authors thank the patients who participated in this study and all individuals who contributed to the establishment of the Center for Cancer Genomics and Advanced Therapeutics database.
