# Supplementary figures and images for: Large-scale Genomic Landscape and Clinical Outcomes of De Novo and Treatment-emergent Neuroendocrine Prostate Cancer
Source: Eur Urol Open Sci. 2026 Jul 2;90:57–68. doi: 10.1016/j.euros.2026.06.003 (PMC13351554; doi:10.1016/j.euros.2026.06.003)

# Supplementary Fig. 1

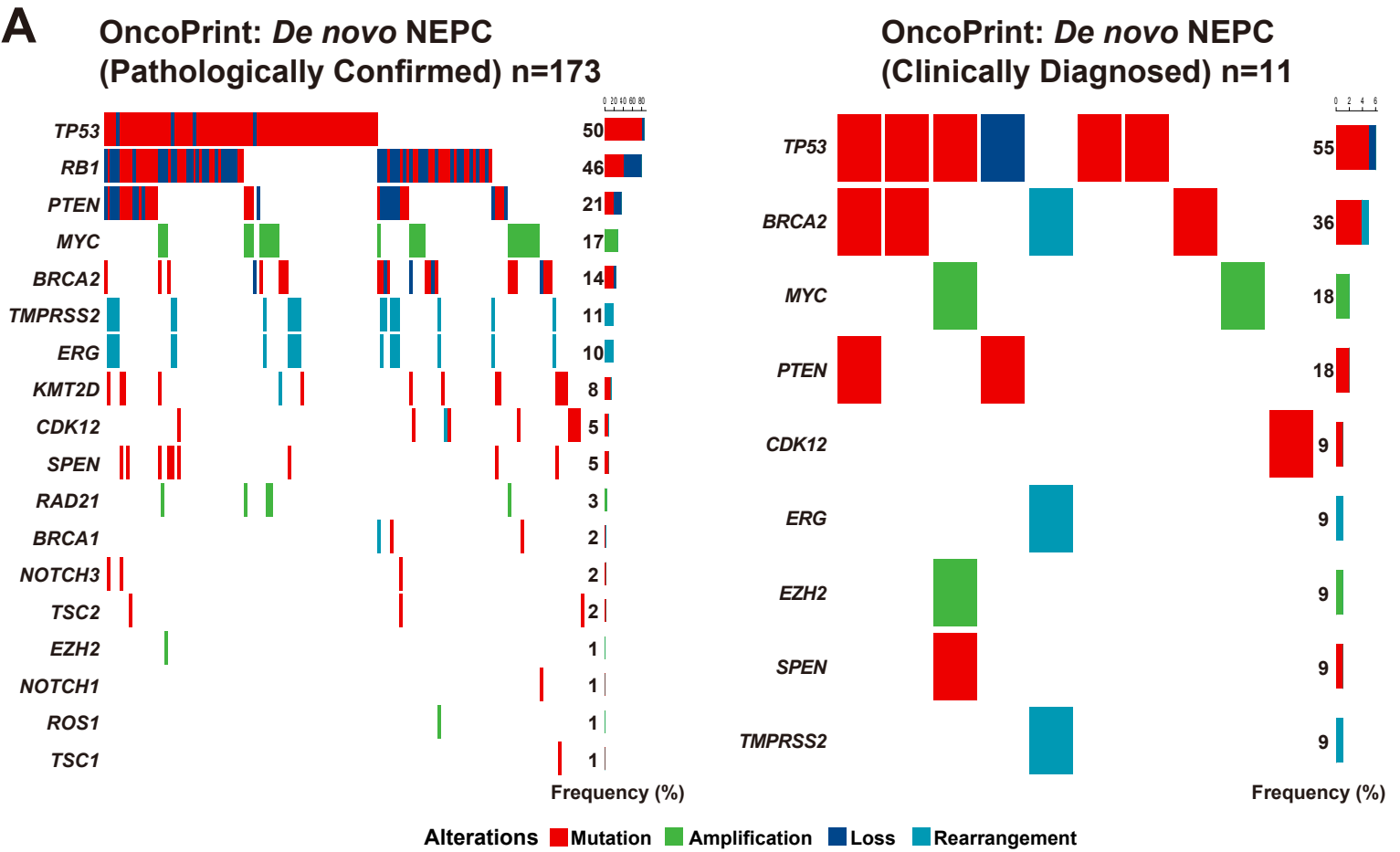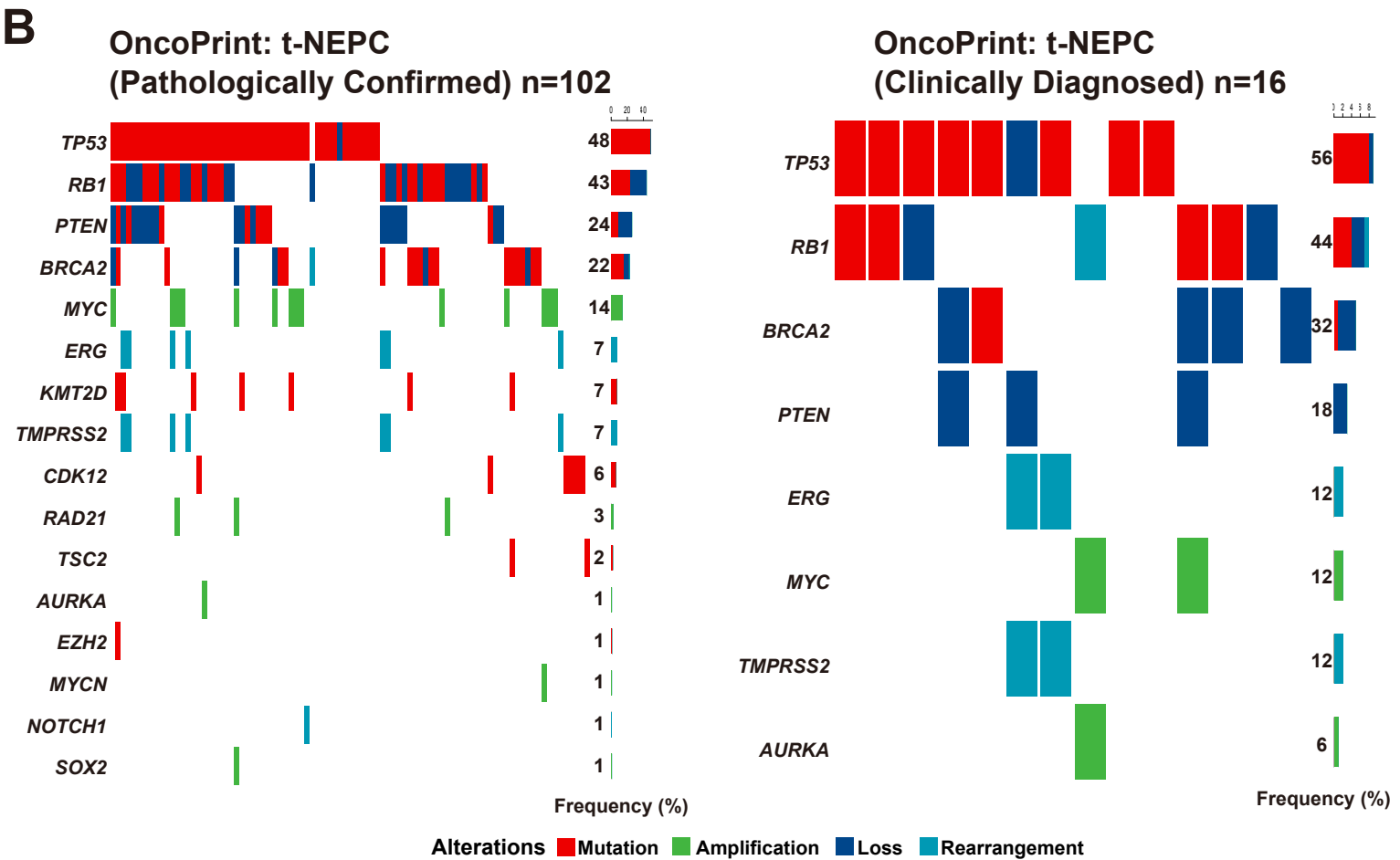

Supplement: Supplementary Data 1 [file mmc1.pdf]

## Supplementary Fig. 2

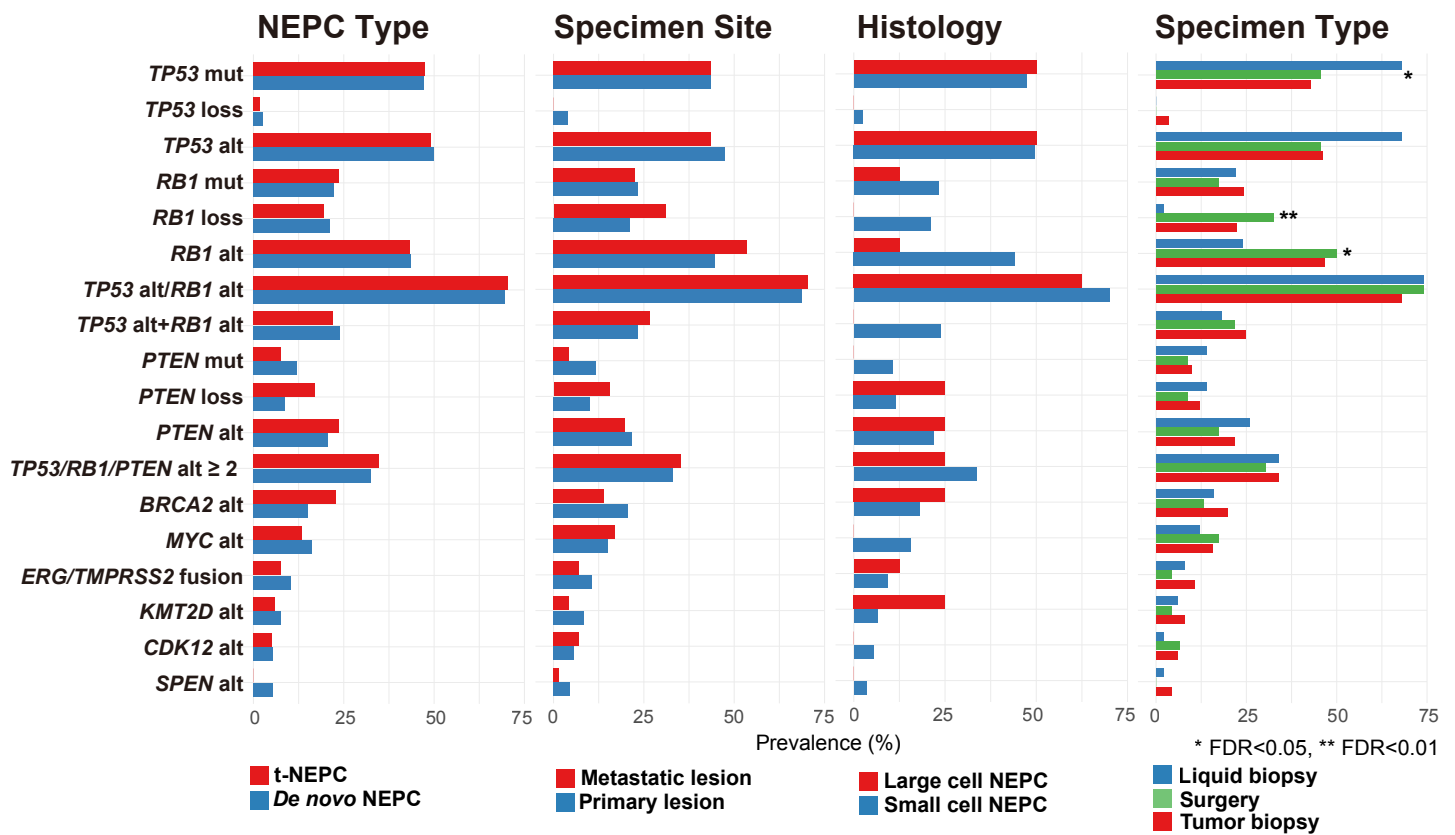

Supplement: Supplementary Data 2 [file mmc2.pdf]

Supplementary Fig. 3

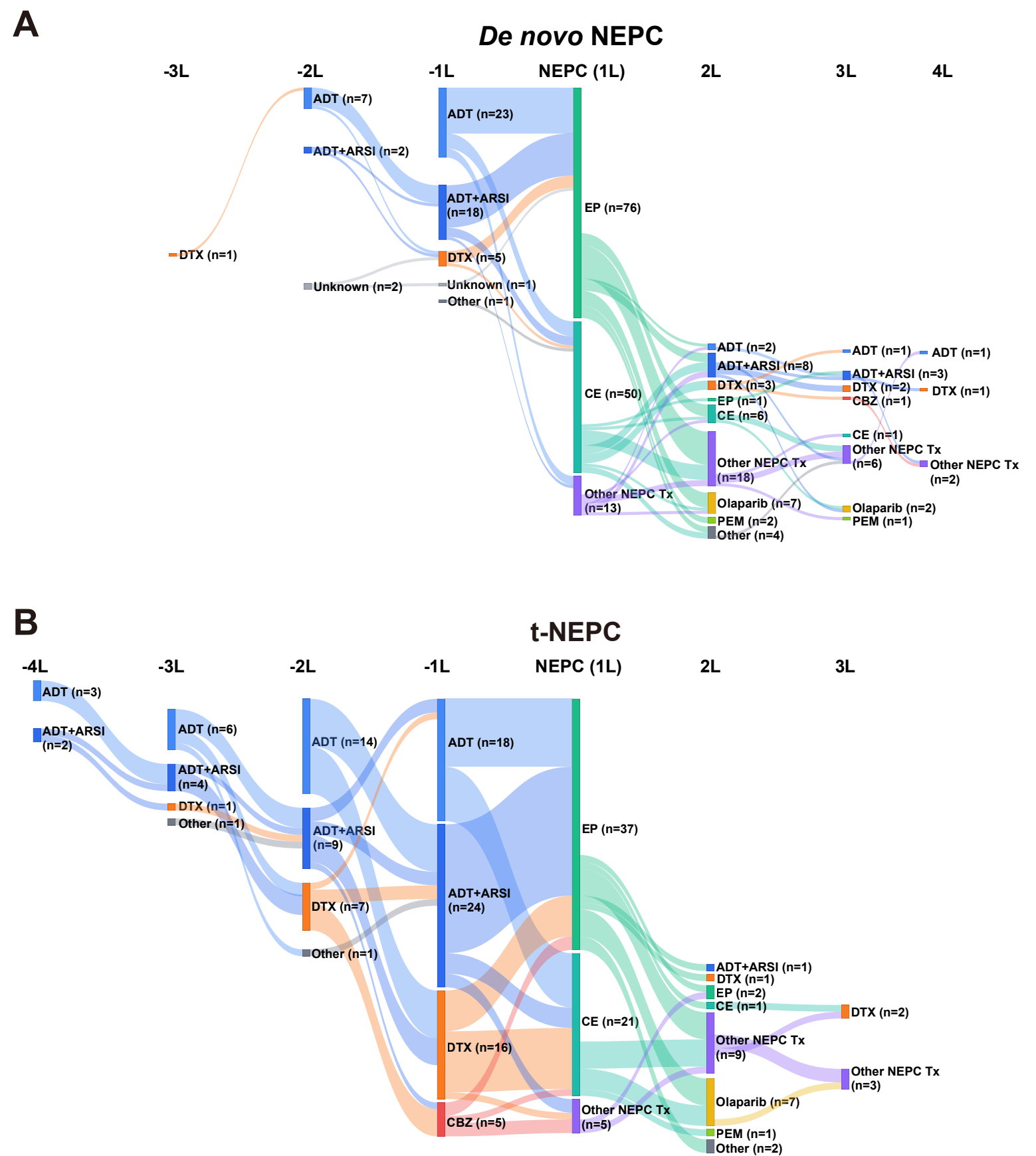

Supplement: Supplementary Data 3 [file mmc3.pdf]

Supplementary Fig. 4

A

De novo NEPC 1

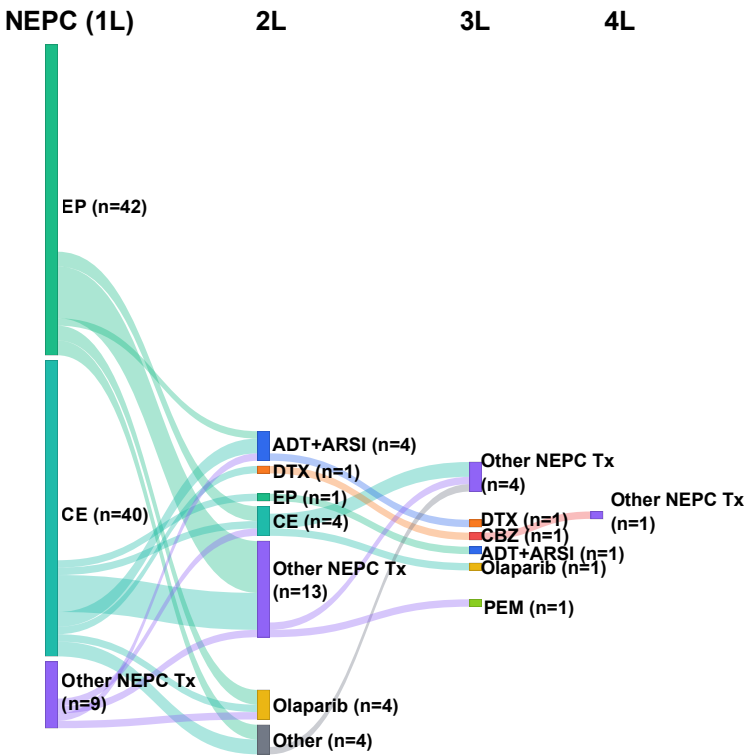

B

De novo NEPC 2

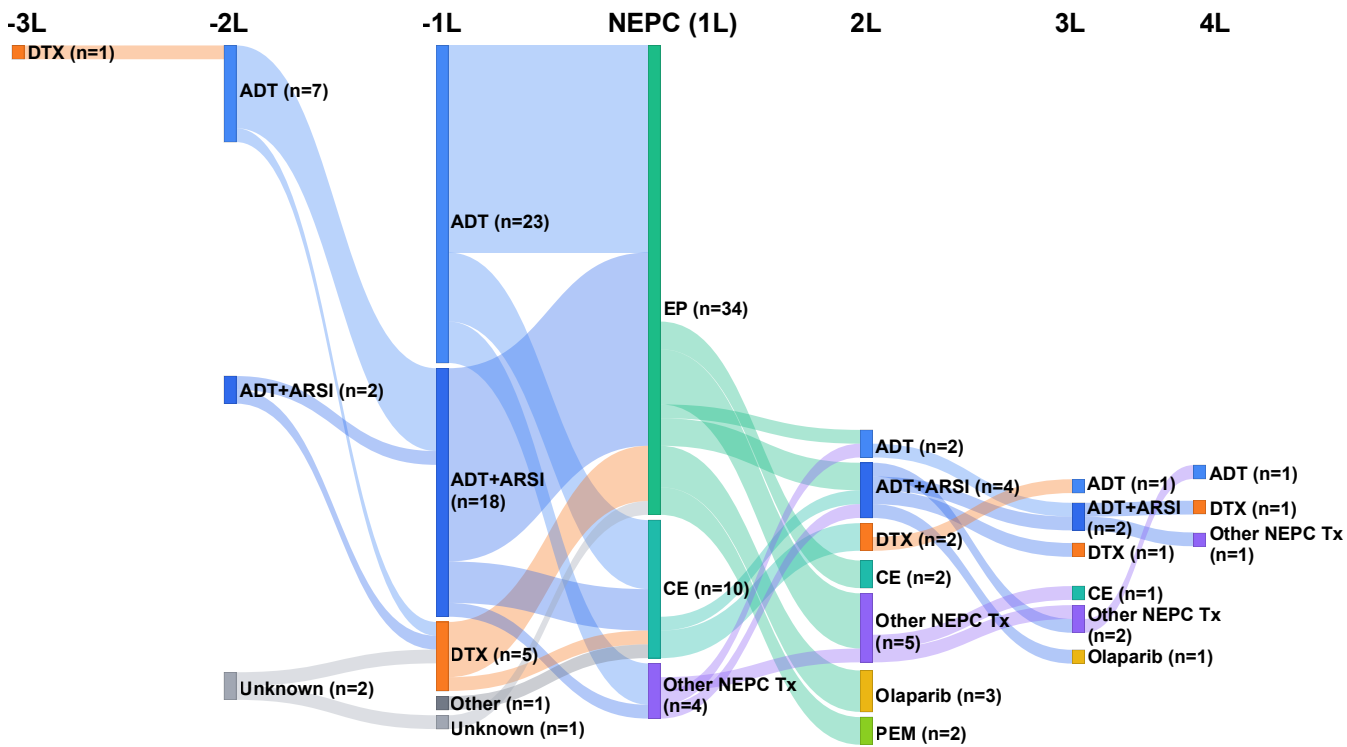

Supplement: Supplementary Data 4 [file mmc4.pdf]

# Supplementary Fig. 5

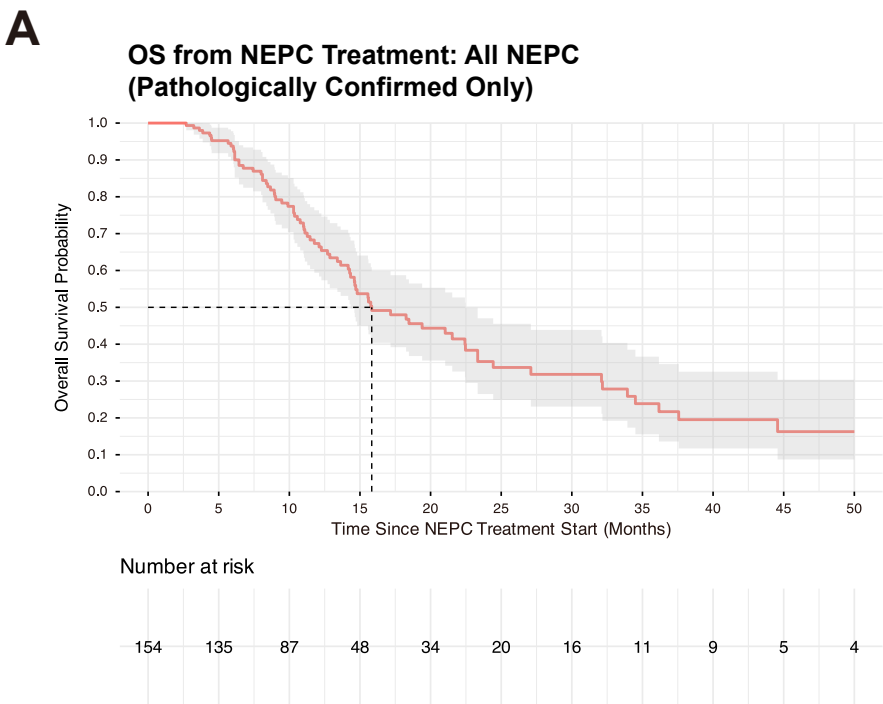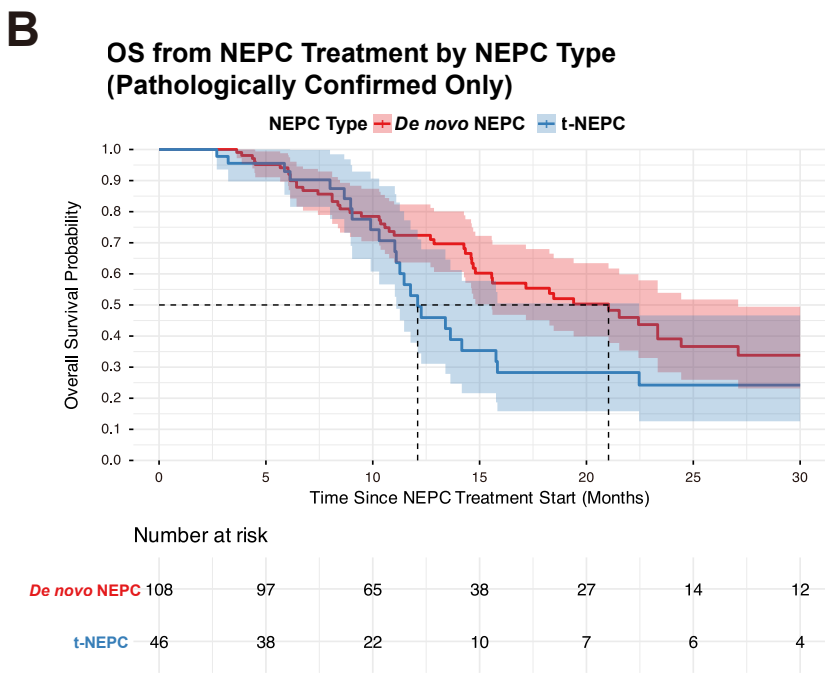

Supplement: Supplementary Data 5 [file mmc5.pdf]

# Supplementary Fig. 6

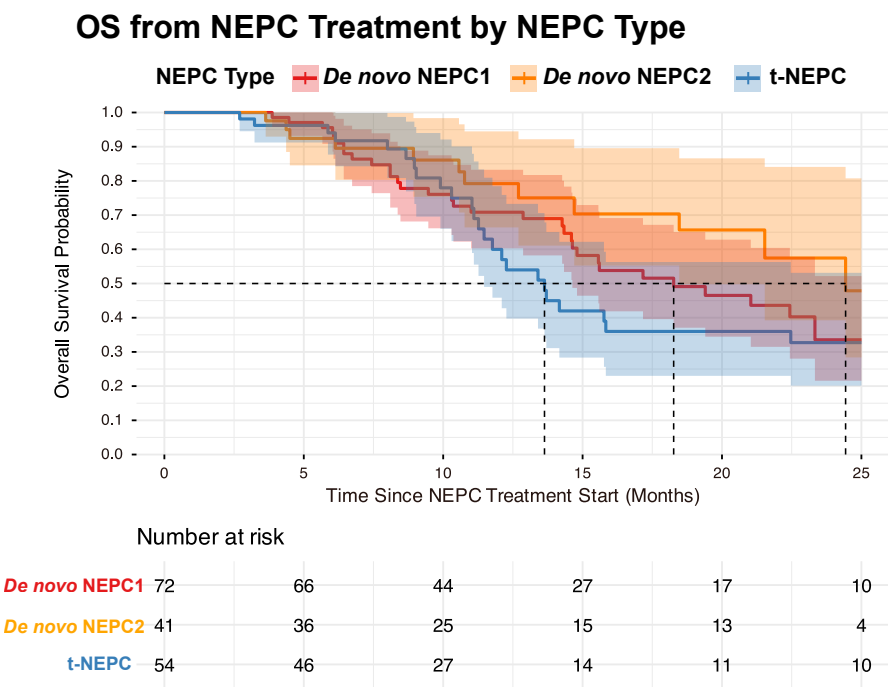

Supplement: Supplementary Data 6 [file mmc6.pdf]
