# Supplementary material for: Large-scale Genomic Landscape and Clinical Outcomes of De Novo and Treatment-emergent Neuroendocrine Prostate Cancer
Source: Eur Urol Open Sci. 2026 Jul 2;90:57–68. doi: 10.1016/j.euros.2026.06.003 (PMC13351554; doi:10.1016/j.euros.2026.06.003)

Supplementary Fig. 7

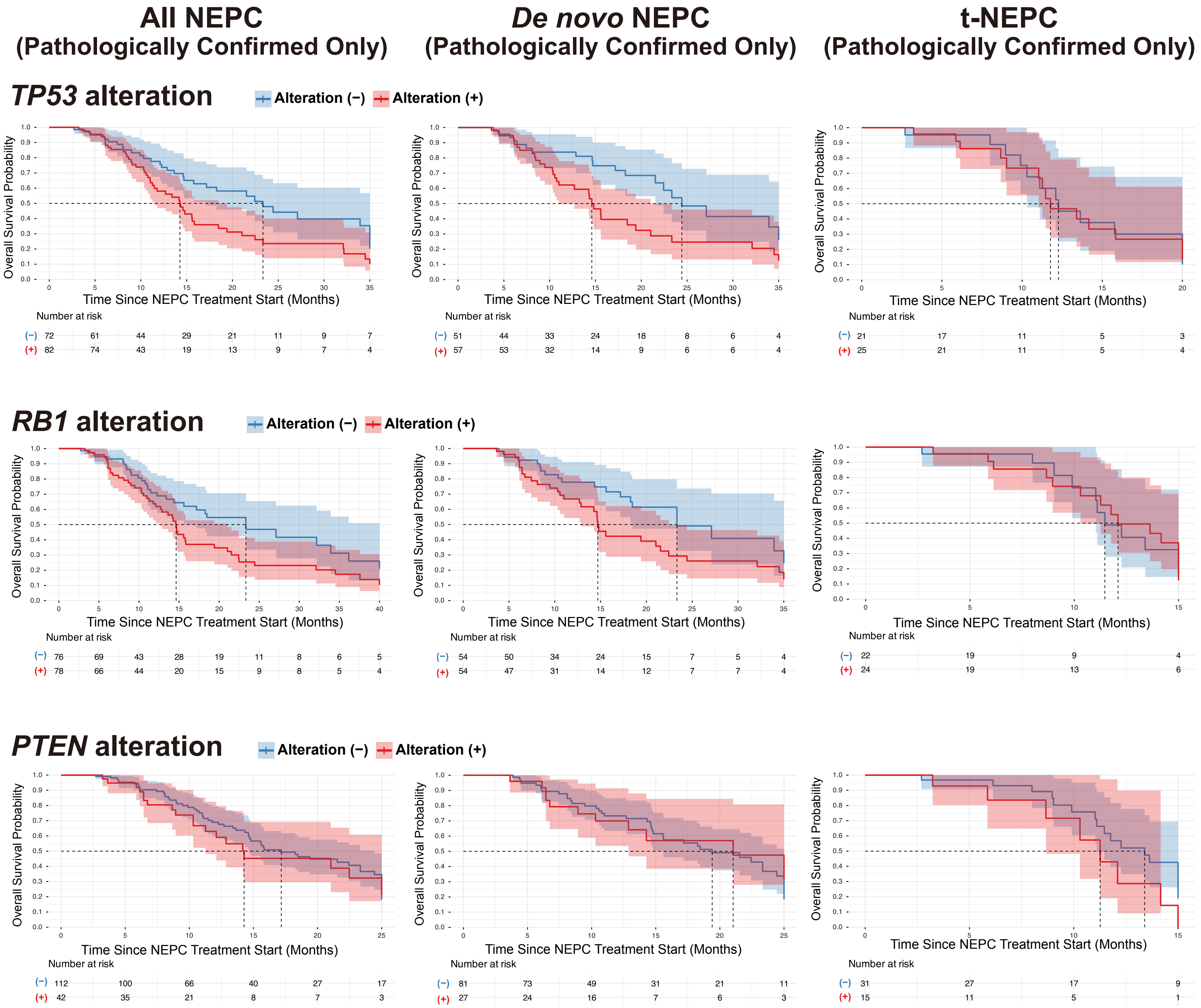

Supplement: Supplementary Data 7 [file mmc7.pdf]
