# Supplementary material for: Large-scale Genomic Landscape and Clinical Outcomes of De Novo and Treatment-emergent Neuroendocrine Prostate Cancer
Source: Eur Urol Open Sci. 2026 Jul 2;90:57–68. doi: 10.1016/j.euros.2026.06.003 (PMC13351554; doi:10.1016/j.euros.2026.06.003)
Supplement: Supplementary Data 9 [file mmc9.docx]

**Supplementary Table 1.**

**Patient characteristics stratified by NEPC type**

| Characteristics | *De novo* NEPC  n = 184 | t-NEPC  n = 118 | p-value^b^  *de novo* NEPC vs  t-NEPC |
| --- | --- | --- | --- |
| Age at Registration^a^ | 72 (67–76) | 72 (67–75) | 0.5 |
| PS |  |  | **0.01** |
| 0 | 99 (54%) | 54 (46%) |  |
| 1 | 64 (35%) | 42 (35%) |  |
| 2≦ | 20 (11%) | 13 (11%) |  |
| Unknown | 1 (<1%) | 9 (8%) |  |
| Metastasis at Registration |  |  | 0.8 |
| Lymph Node Only | 22 (12%) | 16 (14%) |  |
| Bone±Lymph Node | 53 (29%) | 28 (24%) |  |
| Visceral | 83 (45%) | 58 (49%) |  |
| Other | 25 (14%) | 16 (14%) |  |
| CGP testing |  |  | **<0.01** |
| F1Liquid CDx | 20 (11%) | 28 (24%) |  |
| FoundationOne CDx | 147 (80%) | 75 (64%) |  |
| GenMineTOP | 2 (1%) | 3 (2%) |  |
| Guardant360 CDx | 0 (0%) | 2 (2%) |  |
| NCC OncoPanel | 15 (8%) | 10 (8%) |  |
| MTB Timing |  |  | **<0.01** |
| 1L to 2L | 111 (60%) | 31 (26%) |  |
| 3L to 4L | 39 (21%) | 41 (35%) |  |
| 5L or later | 4 (2%) | 12 (10%) |  |
| Unknown | 30 (16%) | 34 (29%) |  |
| Specimen used for MTB |  |  | **<0.01** |
| Surgery | 26 (14%) | 20 (17%) |  |
| Tumor biopsy | 138 (75%) | 68 (58%) |  |
| Liquid biopsy | 20 (11%) | 30 (25%) |  |
| Diagnosis Type |  |  | **0.04** |
| Pathological | 173 (94%) | 102 (86%) |  |
| Clinical | 11 (6%) | 16 (14%) |  |
| FH Breast |  |  | 0.9 |
| Yes | 20 (11%) | 14 (12%) |  |
| FH Ovarian |  |  | 0.15 |
| Yes | 0 (0%) | 2 (2%) |  |
| FH Prostate |  |  | 0.2 |
| Yes | 30 (16%) | 13 (11%) |  |
| FH Pancreas |  |  | 0.8 |
| Yes | 12 (7%) | 9 (8%) |  |

^a^Median (Q1–Q3)

^b^*p*-values (Wilcoxon for continuous; Fisher’s exact for categorical) compare *de novo* NEPC vs t-NEPC.

Bold indicates p < 0.05.

Abbreviations: PS, Performance Status; CGP, Cancer Genome Profiling; MTB, Molecular Tumor Board; FH, family history; NEPC, neuroendocrine prostate cancer; t-NEPC, treatment-emergent neuroendocrine prostate cancer.

**Supplementary Table 2.**

**Patient characteristics of the OS analysis population stratified by NEPC type**

| Characteristics | *De novo* NEPC  n = 113 | t-NEPC  n = 54 | p-value^b^  *de novo* NEPC vs.  t-NEPC |
| --- | --- | --- | --- |
| Age at Registration^a^ | 71 (66–76) | 72 (65–74) | 0.18 |
| PS |  |  | 0.1 |
| 0 | 54 (48%) | 20 (37%) |  |
| 1 | 49 (43%) | 25 (46%) |  |
| 2≦ | 10 (9%) | 7 (13%) |  |
| Unknown | 0 (0%) | 2 (4%) |  |
| Metastasis at Registration |  |  | 0.3 |
| Lymph Node Only | 15 (13%) | 9 (16%) |  |
| Bone±Lymph Node | 25 (22%) | 8 (15%) |  |
| Visceral | 61 (54%) | 35 (65%) |  |
| Other | 12 (11%) | 2 (4%) |  |
| CGP testing |  |  | 0.1 |
| F1Liquid CDx | 14 (12%) | 13 (24%) |  |
| FoundationOne CDx | 89 (79%) | 35 (65%) |  |
| GenMineTOP | 1 (1%) | 2 (4%) |  |
| Guardant360 CDx | 0 (0%) | 0 (0%) |  |
| NCC OncoPanel | 9 (8%) | 4 (7%) |  |
| MTB Timing |  |  | **<0.001** |
| 1L to 2L | 80 (71%) | 15 (28%) |  |
| 3L to 4L | 23 (20%) | 26 (48%) |  |
| 5L or later | 1 (1%) | 7 (13%) |  |
| Unknown | 9 (8%) | 6 (11%) |  |
| Specimen used for MTB |  |  | 0.14 |
| Surgery | 16 (14%) | 8 (15%) |  |
| Tumor biopsy | 83 (73%) | 33 (61%) |  |
| Liquid biopsy | 14 (12%) | 13 (24%) |  |
| Diagnosis Type |  |  | **0.03** |
| Pathological | 108 (96%) | 46 (85%) |  |
| Clinical | 5 (4%) | 8 (15%) |  |
| FH Breast |  |  | 0.5 |
| Yes | 12 (11%) | 8 (15%) |  |
| FH Ovarian |  |  | 0.3 |
| Yes | 0 (0%) | 1 (2%) |  |
| FH Prostate |  |  | 1 |
| Yes | 16 (14%) | 7 (13%) |  |
| FH Pancreas |  |  | 0.5 |
| Yes | 5 (4%) | 4 (7%) |  |

^a^Median (Q1–Q3)

^b^*p*-values (Wilcoxon for continuous; Fisher’s exact for categorical) compare *de novo* NEPC vs. t-NEPC.

Bold indicates *p* < 0.05.

Abbreviations: PS, Performance Status; CGP, Cancer Genome Profiling; MTB, Molecular Tumor Board; FH, family history; NEPC, neuroendocrine prostate cancer; t-NEPC, treatment-emergent neuroendocrine prostate cancer.
