# Supplementary material for: Large-scale Genomic Landscape and Clinical Outcomes of De Novo and Treatment-emergent Neuroendocrine Prostate Cancer
Source: Eur Urol Open Sci. 2026 Jul 2;90:57–68. doi: 10.1016/j.euros.2026.06.003 (PMC13351554; doi:10.1016/j.euros.2026.06.003)
Supplement: Supplementary Data 10 [file mmc10.pdf]

Supplementary Table 3. Survival estimates and unadjusted HRs for OS from initiation of NEPC treatment in all patients and according to NEPC type (See also Figure 3).

	Restricted follow-up		Full follow-up
	**Cutoff (Mo)**	**mOS (Mo) (95% CI)**	**mOS (Mo) (95% CI)**
All NEPC	50	18.5 (14.7-24.4)	18.5 (14.7-24.4)

**Analysis population**	**Restricted follow-up**					**Full follow-up**		
	**Cutoff (Mo)**	**NEPC Type**	**mOS (Mo) (95% CI)**	**HR (95% CI)**	**Log-rank p-value**	**mOS (Mo) (95% CI)**	**HR (95% CI)**	**Log-rank p-value**
By NEPC Type	40	*De novo* NEPC	21.5 (15.6-32.1)	1.29 (0.81-2.07)	0.3	21.5 (15.6-32.1)	1.36 (0.86-2.16)	0.2
		t-NEPC	13.6 (11.5-32.2)			13.6 (11.5-32.2)		

Hazard ratios compare t-NEPC with *de novo* NEPC and were estimated using univariable Cox proportional hazards models. p values correspond to the log-rank test. Restricted follow-up analyses used administrative censoring at the prespecified truncation time point.

Mo, month; NEPC, neuroendocrine prostate cancer; t-NEPC, treatment-emergent neuroendocrine prostate cancer; OS, overall survival; HR, hazard ratio; CI, confidence interval; mOS, median overall survival.

Supplementary Table 4. Survival estimates and unadjusted HRs for OS from initiation of NEPC treatment according to *TP53*, *RB1*, and *PTEN* alteration status (See also Figure 4).

Analysis population	Restricted follow-up						Full Follow-up				
	Cutoff (Mo)	NEPC Type	Alteration status	mOS (Mo) (95% CI)	HR (95% CI)	Log-rank p-value	NEPC Type	Alteration status	mOS (Mo) (95% CI)	HR (95% CI)	Log-rank p-value
TP53 alteration	45	All NEPC	-	24.4 (18.3-37.6)	1.58 (1.01-2.48)	**0.04**	All NEPC	-	24.4 (18.3-37.6)	1.58 (1.01-2.48)	**0.04**
			+	14.6 (12.7-21.0)				+	14.6 (12.7-21.0)		
	60	*De novo* NEPC	-	24.4 (22.4-NA)	2.00 (1.13-3.55)	**0.02**	*De novo* NEPC	-	24.4 (22.4-NA)	1.99 (1.12-3.53)	**0.02**
			+	14.8 (12.7-23.3)				+	14.8 (12.7-23.3)		
	20	t-NEPC	-	12.3 (11.1-NA)	0.82 (0.39-1.7)	0.7	t-NEPC	-	12.3 (11.1-NA)	1.11 (0.52-2.37)	0.8
			+	13.7 (11.5-20.0)				+	13.7 (11.5-NA)		
RB1 alteration	40	All NEPC	-	27.1 (18.3-40.0)	1.72 (1.11-2.69)	**0.01**	All NEPC	-	27.1 (18.3-46.2)	1.72 (1.10-2.68)	**0.02**
			+	14.6 (12.9-21.0)				+	14.6 (12.9-21.0)		
	35	*De novo* NEPC	-	27.1 (23.3-NA)	1.93 (1.10-3.40)	**0.02**	*De novo* NEPC	-	27.1 (23.3-NA)	1.92 (1.09-3.37)	**0.02**
			+	14.7 (12.9-22.4)				+	14.7 (12.9-22.4)		
	20	t-NEPC	-	13.4 (11.1-NA)	1.20 (0.58-2.50)	0.6	t-NEPC	-	13.4 (11.1-NA)	1.50 (0.69-3.26)	0.3
			+	13.6 (11.3-20.0)				+	13.6 (11.3-NA)		
PTEN alteration	25	All NEPC	-	19.4 (15.6-25.0)	1.33 (0.79-2.22)	0.3	All NEPC	-	19.4 (15.6-32.1)	1.37 (0.82-2.31)	0.2
			+	14.3 (11.3-NA)				+	14.3 (11.3-NA)		
	25	*De novo* NEPC	-	21.5 (15.6-25.0)	0.99 (0.51-1.92)	0.98	*De novo* NEPC	-	21.5 (15.6-32.1)	1.01 (0.51-1.97)	0.98
			+	21.0 (12.9-NA)				+	21.0 (12.9-NA)		
	15	t-NEPC	-	13.7 (11.8-15.0)	2.45 (1.05-5.71)	**0.04**	t-NEPC	-	13.7 (11.8-NA)	2.64 (1.11-6.27)	**0.02**
			+	11.3 (8.7-NA)				+	11.3 (8.7-NA)		

Hazard ratios compare patients with alterations (+) with those without alterations (−) and were estimated using univariable Cox proportional hazards models. p values correspond to the log-rank test. Restricted follow-up analyses used administrative censoring at the prespecified truncation time point.

Mo, month; NEPC, neuroendocrine prostate cancer; t-NEPC, treatment-emergent neuroendocrine prostate cancer; OS, overall survival; HR, hazard ratio; CI, confidence interval; mOS, median overall survival; NR, not reached.

Supplementary Table 5. Survival estimates and unadjusted HRs for OS from initiation of NEPC treatment according to *TP53/RB1/PTEN* alteration group (See also Figure 5).

**Analysis population**	**Restricted follow-up**						**Full follow-up**			
	**Alteration status**	**Cutoff (Mo)**	**mOS (Mo) (95% CI)**	**Global log-rank p-value**	**HR (95% CI)**	**Cox p-value**	**mOS (Mo) (95% CI)**	**Global log-rank p-value**	**HR (95% CI)**	**Cox p-value**
All NEPC	WT (0 Alt)	15	15.0 (15.0-NA)	**0.003**	Reference	-	33.9 (23.3-NA)	**0.005**	Reference	-
	Alt 1–2		15.0 (14.3-15.0)		1.51 (0.86-2.65)	0.156	15.8 (14.3-24.4)		1.41 (0.80-2.48)	0.235
	Alt 3		14.2 (8.7-NA)		3.47 (1.67-7.23)	**<0.001**	14.2 (8.7-NA)		3.13 (1.51-6.48)	**0.002**
De novo NEPC	WT (0 Alt)	15	NR (NA-NA)	**0.01**	Reference	-	27.1 (23.3-NA)	**0.043**	Reference	-
	Alt 1–2		15.0 (14.6-15.0)		2.13 (1.01-4.46)	**0.046**	18.5 (14.6-32.1)		1.79 (0.85-3.74)	0.124
	Alt 3		14.3 (6.7-NA)		4.40 (1.65-11.71)	**0.003**	14.3 (6.7-NA)		3.31 (1.26-8.68)	**0.015**
t-NEPC	WT (0 Alt)	10	10.0 (9.9-NA)	**0.03**	Reference	-	12.3 (9.9-NA)	0.096	Reference	-
	Alt 1–2		10.0 (10.0-NA)		0.58 (0.24-1.41)	0.23	13.7 (12.1-NA)		1.04 (0.39-2.73)	0.942
	Alt 3		10.0 (5.9-NA)		1.72 (0.58-5.14)	0.331	11.3 (5.9-NA)		2.75 (0.83-9.08)	0.097

Hazard ratios and Cox p-values were estimated using univariable Cox proportional hazards models with WT (0 Alt) as the reference group. Global p-values correspond to log-rank tests across alteration groups. Restricted follow-up analyses used administrative censoring at the prespecified truncation time point.

NEPC, neuroendocrine prostate cancer; t-NEPC, treatment-emergent neuroendocrine prostate cancer; OS, overall survival; HR, hazard ratio; CI, confidence interval; WT, wild type; Alt, alteration; Mo, month; mOS, median overall survival; NR, not reached.

Supplementary Table 6. Multivariable Cox proportional hazards models for OS from initiation of NEPC treatment according to TP53/RB1/PTEN alteration group (See also Figure 5).

Analysis population	Restricted follow-up						Full follow-up		
	Cutoff (Mo)	Variable	Category	N	Adjusted HR (95% CI)	Cox p-value	N	Adjusted HR (95% CI)	Cox p-value
All NEPC	15	*TP53/RB1/PTEN* alteration group	WT (0 Alt)	37	Reference	—	37	Reference	—
			Alt 1–2	109	1.68 (0.76–3.74)	0.2	109	1.24 (0.68–2.26)	0.5
			Alt 3	21	3.08 (1.20–7.88)	**0.02**	21	2.43 (1.13–5.22)	**0.02**
De novo NEPC	15	*TP53/RB1/PTEN* alteration group	WT (0 Alt)	25	Reference	—	25	Reference	—
			Alt 1–2	75	3.32 (0.97–11.41)	0.06	75	1.73 (0.79–3.78)	0.17
			Alt 3	13	4.97 (1.17–21.13)	**0.03**	13	2.58 (0.92–7.19)	0.07
t-NEPC	10	*TP53/RB1/PTEN* alteration group	WT (0 Alt)	12	Reference	—	12	Reference	—
			Alt 1–2	34	0.33 (0.07–1.69)	0.18	34	0.68 (0.23–2.00)	0.5
			Alt 3	8	1.23 (0.24–6.43)	0.8	8	2.04 (0.50–8.31)	0.3

Hazard ratios and p values were estimated using multivariable Cox proportional hazards models adjusted for age at registration, performance status at registration, and metastatic pattern at registration. WT (0 Alt) was used as the reference group.

Restricted follow-up analyses used administrative censoring at the prespecified truncation time point.

Mo, month; NEPC, neuroendocrine prostate cancer; t-NEPC, treatment-emergent neuroendocrine prostate cancer; OS, overall survival; HR, hazard ratio; CI, confidence interval.

Supplementary Table 7. Cox proportional hazards models including interaction terms between NEPC type and genomic alterations for OS from initiation of NEPC treatment (See also Figure 5).

Exposure	Restricted follow-up			Full follow-up	
	Cutoff (Mo)	Adjusted Interaction HR (95% CI)	Interaction p-value	Adjusted Interaction HR (95% CI)	Interaction p-value
TP53 alteration × NEPC type	45	0.41 (0.15–1.11)	0.08	0.42 (0.16–1.11)	0.08
RB1 alteration × NEPC type	40	1.38 (0.51–3.77)	0.5	1.19 (0.45–3.17)	0.7
PTEN alteration × NEPC type	25	3.32 (1.04–10.61)	**0.043**	2.88 (0.95–8.76)	0.06
TP53/*RB1*/*PTEN* Alt 1–2 × NEPC type	15	0.27 (0.05–1.38)	0.1	0.64 (0.19–2.15)	0.5
TP53/*RB1*/*PTEN* Alt 3 × NEPC type	15	0.48 (0.07–3.37)	0.5	0.96 (0.20–4.48)	0.95

Interaction hazard ratios were estimated using Cox proportional hazards models adjusted for age at registration, performance status at registration, and metastatic pattern at registration. Restricted follow-up analyses used administrative censoring at the prespecified truncation time point.

Mo, month; NEPC, neuroendocrine prostate cancer; OS, overall survival; HR, hazard ratio; CI, confidence interval.

Supplementary Table 8. Survival estimates and unadjusted HRs for OS from initiation of NEPC treatment in patients with pathologically confirmed NEPC, overall and by NEPC type (See also Supplementary Figure 5).

Analysis population	Restricted follow-up		Full follow-up
	Cutoff (Mo)	mOS (Mo) (95% CI)	mOS (Mo) (95% CI)
All pathologically confirmed NEPC	50	15.8 (14.3-22.5)	15.8 (14.3-22.5)

Analysis population	Restricted follow-up					Full follow-up		
	Cutoff (Mo)	NEPC Type	mOS (Mo) (95% CI)	HR (95% CI)	Log-rank p-value	mOS (Mo) (95% CI)	HR (95% CI)	Log-rank p-value
By NEPC type<br>Pathologically confirmed NEPC	30	*De novo* NEPC	21.0 (15.6-27.1)	1.53 (0.92-2.52)	0.1	21.0 (15.6-27.1)	1.56 (0.97-2.51)	0.06
		t-NEPC	12.1 (11.1-22.5)			12.1 (11.1-22.5)		

Hazard ratios compare t-NEPC with *de novo* NEPC and were estimated using univariable Cox proportional hazards models. p values correspond to the log-rank test. Analyses were restricted to patients with pathologically confirmed NEPC. Restricted follow-up analyses used administrative censoring at the prespecified truncation time point.

Mo, month; NEPC, neuroendocrine prostate cancer; t-NEPC, treatment-emergent neuroendocrine prostate cancer; OS, overall survival; HR, hazard ratio; CI, confidence interval; mOS, median overall survival.

Supplementary Table 9. Survival estimates and unadjusted HRs for OS from initiation of NEPC treatment in all patients and according to NEPC type (See also Supplementary Figure 6).

Analysis population	Restricted follow-up					Full follow-up		
	Cutoff (Mo)	NEPC Type	mOS (Mo) (95% CI)	HR (95% CI)	Global log-rank p-value	mOS (Mo) (95% CI)	HR (95% CI)	Global log-rank p-value
By NEPC Type	25	*De novo* NEPC1	18.3 (14.6-NR)	Reference	0.2	18.3 (14.6-33.9)	Reference	0.4
		De novo NEPC2	24.4 (18.5-NR)	0.64 (0.33-1.23)		24.4 (18.5-NR)	0.81 (0.44-1.49)	
		t-NEPC	13.6 (11.5-NR)	1.18 (0.70-2.00)		13.6 (11.5–32.2)	1.32 (0.80-2.15)	

Hazard ratios compare de novo NEPC2 and t-NEPC with de novo NEPC1 and were estimated using univariable Cox proportional hazards models. p values correspond to global log-rank tests across NEPC type groups. Restricted follow-up analyses used administrative censoring at the prespecified truncation time point.

Mo, month; NEPC, neuroendocrine prostate cancer; t-NEPC, treatment-emergent neuroendocrine prostate cancer; OS, overall survival; HR, hazard ratio; CI, confidence interval; mOS, median overall survival.

Supplementary Table 10. Survival estimates and unadjusted HRs for OS from initiation of NEPC treatment according to TP53, RB1, and PTEN alteration status in patients with pathologically confirmed NEPC (See also Supplementary Figure 7).

Analysis population	Restricted follow-up						Full follow-up				
	**Cutoff (Mo)**	**NEPC Type**	**Alteration status**	**mOS (Mo) (95% CI)**	**HR (95% CI)**	**Log-rank p-value**	**NEPC Type**	**Alteration status**	**mOS (Mo) (95% CI)**	**HR (95% CI)**	**Log-rank p-value**
TP53 alteration	35	All NEPC	-	23.3 (17.2-35.0)	1.74 (1.1-2.75)	**0.02**	All NEPC	-	23.3 (17.2-37.6)	1.72 (1.08-2.72)	**0.02**
			+	14.3 (11.5-18.5)				+	14.3 (11.5-18.5)		
	35	*De novo* NEPC	-	24.4 (21.5-NA)	2.03 (1.14-3.61)	**0.01**	*De novo* NEPC	-	24.4 (21.5-NA)	2.02 (1.13-3.58)	**0.01**
			+	14.6 (11.0-21.0)				+	14.6 (11.0-21.0)		
	20	t-NEPC	-	12.3 (10.3-NA)	1.02 (0.47-2.21)	0.9	t-NEPC	-	12.3 (10.3-NA)	1.33 (0.59-3.02)	0.5
			+	11.8 (11.0-20.0)				+	11.8 (11.0-NA)		
RB1 alteration	40	All NEPC	-	23.3 (17.2-40.0)	1.62 (1.03-2.56)	**0.04**	All NEPC	-	23.3 (17.2-NA)	1.61 (1.02-2.54)	**0.04**
			+	14.6 (12.7-21.0)				+	14.6 (12.7-21.0)		
	35	*De novo* NEPC	-	23.3 (18.5-NA)	1.75 (1.00-3.08)	**0.048**	*De novo* NEPC	-	23.3 (18.5-NA)	1.74 (0.99-3.06)	0.051
			+	14.7 (12.9-22.4)				+	14.7 (12.9-22.4)		
	15	t-NEPC	-	13.4 (11.1-NA)	1.20 (0.58-2.50)	0.6	t-NEPC	-	13.4 (11.1-NA)	1.50 (0.69-3.26)	0.3
			+	13.6 (11.3-20.0)				+	13.6 (11.3-NA)		
PTEN alteration	25	All NEPC	-	17.2 (14.6-24.4)	1.19 (0.71-2.00)	0.5	All NEPC	-	17.2 (14.6-24.4)	1.21 (0.72-2.05)	0.5
			+	14.3 (11.3-NA)				+	14.3 (11.3-NA)		
	25	*De novo* NEPC	-	19.4 (15.6-25.0)	0.92 (0.47-1.80)	0.8	*De novo* NEPC	-	19.4 (15.6-27.1)	0.94 (0.48-1.84)	0.9
			+	21.0 (12.9-NA)				+	21.0 (12.9-NA)		
	15	t-NEPC	-	13.4 (11.1-15.0)	2.01 (0.85-4.73)	0.1	t-NEPC	-	13.4 (11.1-NA)	2.08 (0.87-4.96)	0.1
			+	11.3 (8.7-NA)				+	11.3 (8.7-NA)		

Hazard ratios compare patients with alterations (+) with those without alterations (−) and were estimated using univariable Cox proportional hazards models. p values correspond to the log-rank test. Analyses were restricted to patients with pathologically confirmed NEPC. Restricted follow-up analyses used administrative censoring at the prespecified truncation time point.

Mo, month; NEPC, neuroendocrine prostate cancer; t-NEPC, treatment-emergent neuroendocrine prostate cancer; OS, overall survival; HR, hazard ratio; CI, confidence interval; mOS, median overall survival; NR, not reached.

Supplementary Table 11. Survival estimates and unadjusted HRs for OS from initiation of NEPC treatment according to TP53/RB1/PTEN alteration group in patients with pathologically confirmed NEPC (See also Supplementary Figure 8).

Analysis population	Restricted follow-up					Full follow-up		
	Alteration status	Cutoff (Mo)	mOS (Mo) (95% CI)	HR (95% CI)	Global log-rank p-value	mOS (Mo) (95% CI)	HR (95% CI)	Global log-rank p-value
All NEPC	WT (0 Alt)	15	15.0 (15.0-NA)	Reference	**0.009**	27.1 (18.3-NA)	Reference	**0.014**
	Alt 1–2		15.0 (13.6-15.0)	1.52 (0.85–2.73)		15.6 (13.6-22.4)	1.44 (0.81–2.59)	
	Alt 3		14.2 (8.7-NA)	3.12 (1.43–6.82)		14.2 (8.7-NA)	2.84 (1.31–6.16)	
De novo NEPC	WT (0 Alt)	15	NR (NA-NA)	Reference	**0.019**	27.1 (23.3-NA)	Reference	0.07
	Alt 1–2		15.0 (14.6-15.0)	2.02 (0.96–4.24)		15.6 (14.6-24.4)	1.74 (0.83–3.65)	
	Alt 3		14.3 (6.7-NA)	4.10 (1.42–11.81)		14.3 (6.7-NA)	2.82 (1.03–7.74)	
t-NEPC	WT (0 Alt)	10	10.0 (9.9-NA)	Reference	0.09	11.1 (9.9-NA)	Reference	0.3
	Alt 1–2		10.0 (10.0-NA)	0.66 (0.25–1.72)		13.4 (11.5-NA)	1.04 (0.36–2.99)	
	Alt 3		10.0 (5.9-NA)	1.61 (0.52–5.02)		11.3 (5.9-NA)	1.70 (0.51–5.59)	

Hazard ratios were estimated using univariable Cox proportional hazards models with WT (0 Alt) as the reference group. p values correspond to global log-rank tests across alteration groups. Analyses were restricted to patients with pathologically confirmed NEPC. Restricted follow-up analyses used administrative censoring at the prespecified truncation time point.

Mo, month; NEPC, neuroendocrine prostate cancer; t-NEPC, treatment-emergent neuroendocrine prostate cancer; OS, overall survival; HR, hazard ratio; CI, confidence interval; WT, wild type; Alt, alteration; mOS, median overall survival; NR, not reached.
